# Mukara: A deep learning alternative to the four-step travel demand model with a case study on interurban highway traffic prediction in the UK

**DOI:** 10.1371/journal.pone.0345576

**Published:** 2026-04-16

**Authors:** Yue Li, Shujuan Chen, Ying Jin

**Affiliations:** Martin Centre for Architectural and Urban Studies, University of Cambridge, Cambridge, United Kingdom; University of Salerno: Universita degli Studi di Salerno, ITALY

## Abstract

Accurate traffic volume prediction is essential for managing congestion, improving road safety, mitigating environmental impacts, and supporting long-term transportation planning. The traditional four-step travel demand model (FSM) is a well-established framework, but it relies on static survey data, substantial calibration effort, and simplified behavioural assumptions that may not adequately capture complex travel patterns. In contrast, data-driven models are capable of learning nonlinear relationships from large datasets, yet they are often designed for short-term forecasting and typically do not target the long-term, segment-level volume estimation tasks required for strategic planning. This study proposes Mukara, a deep learning framework that directly approximates the mapping from external socioeconomic and network features to observed traffic volumes on highway trunk road segments. The model is trained on eight years of data from England and Wales and incorporates population, employment, land use, road network characteristics, and points of interest as inputs. Mukara achieves a mean GEH of 50.74, a mean absolute error of 8,989 vehicles per day, and an *R*^2^ of 0.583 under random cross-validation, outperforming baseline models and existing studies under comparable settings. Under a more stringent region-based spatial cross-validation scheme, performance remains robust, demonstrating strong spatial transferability. Ablation experiments further demonstrate the robustness of the proposed architecture and reveal the relative importance of different input feature groups for prediction.

## Introduction

Traffic prediction plays a pivotal role in addressing critical challenges such as reducing congestion, mitigating carbon emissions and pollution, and improving road safety and public health [1–5]. In the United Kingdom, road transport is the predominant mode of travel, accounting for 86% of all passenger kilometres in 2022 [6]. This trend is consistent with many OECD countries, where road transport similarly dominates passenger travel [7]. Simultaneously, vehicle ownership is rapidly increasing in the Global South, with projections indicating that by 2030, 56% of the world’s vehicles will be owned by non-OECD countries, compared to 24% in 2002 [8]. A robust traffic prediction system can help travellers plan routes effectively, assist traffic operators in informed decision-making, and enhance overall traffic management efficiency [9].

Despite advancements in traffic prediction, research has predominantly focused on urban traffic, leaving interurban traffic networks relatively underexplored [10]. Interurban settings, however, present a unique opportunity for testing novel traffic prediction models due to the abundance of high-quality data and the relatively lower complexity of traffic patterns compared to urban areas. Urban traffic is often influenced by localised factors such as pedestrian activity, public transit systems, and highly variable demand patterns, making it noisier and more challenging to model. In contrast, interurban traffic data typically exhibits more stable and predictable patterns, making it ideal for evaluating the feasibility of innovative approaches like Mukara.

As shown in Table 1, traffic prediction has traditionally relied on two main approaches: the four-step travel demand model (FSM) and deep learning-based models. The FSM has long served as a foundational framework in transportation planning [11]. It decomposes travel behaviour into trip generation and attraction, trip distribution, mode choice, and traffic assignment. A concise overview of the FSM structure and its sequential components is provided in Appendix S1 (S1 File). In principle, the FSM provides a clear mapping from zonal socioeconomic inputs to an origin-destination (OD) matrix and then to link-level flows. Its modular structure and behavioural grounding have made it a cornerstone of planning practice for decades. In practice, however, FSM implementations often rely on restrictive functional forms and oversimplified assumptions. The sequential structure of the FSM also treats its steps as largely independent, even though destination choice, mode choice, and route choice are closely interrelated in reality [12]. As a result, FSM-based workflows typically require repeated calibration of the OD matrix and assignment parameters, which is labour-intensive and increasingly difficult at large spatial scales or under novel planning scenarios [13]. A case study in Istanbul found huge discrepancies between daily traffic predicted by FSM and the actual observed daily traffic volume [14].

**Table 1:** A comparison between the four-step travel demand model and existing deep learning models.

	The four-step model	Deep learning models
**Model**	A rule-based model based on planning assumptions	Empirical model that extrapolates complex patterns from historical data; often highly accurate and detailed
**Inputs**	Population, household size, employment, land use characteristics, travel costs, and other relevant features.	Mainly historical sensor readings from the traffic network, sometimes with external variables as auxiliary inputs
**Outputs**	Trip generation and attraction rates, origin–destination (OD) flow matrices, modal split distributions, and long-term traffic volume forecasts	Immediate or short-term prediction of traffic in the next 5, 15, 60 minutes (nowcasting)
**Limitations**	Low accuracy and resolution in its predictions due to reliance on simplified assumptions and rules	Black-box models that rely heavily on historical inputs, cannot predict locations without sensors, have limited scalability and transferability, and provide little insight into traffic determinants

On the other hand, deep learning models have emerged as powerful alternatives due to their ability to model complex spatial-temporal dependencies in traffic data [10,15]. Architectures such as Convolutional Neural Networks (CNNs) [16], Recurrent Neural Networks (RNNs) [17], Long Short-Term Memory networks (LSTMs) [18], and Gated Recurrent Units (GRUs) [19] have significantly improved the modelling of temporal trends, while recent advancements like Transformers [20], Graph Neural Networks (GNNs) [21], and Graph Attention Networks (GATs) [22] further enable spatial reasoning within graph-structured networks. State-of-the-art models such as ST-ResNet [23], DCRNN [24], ConvLSTM [25], PFNet [26], and STGAT [27] incorporate these techniques to produce highly accurate short-term predictions. However, most of these models treat traffic prediction as a time-series forecasting task based on historical sensor readings. As such, their outputs often reflect patterns seen in the past rather than insights into the determinants of traffic dynamics. This is illustrated in recent work such as ST-MetaNet [28,29], where the predictions strongly mirror input trends. While some models incorporate external features such as points of interest (POIs), road types, and event data [23,30], these are typically used as auxiliary inputs rather than as primary determinants.

Motivated by these limitations, recent studies have explored machine learning models as alternatives for individual steps of the FSM. Deep learning–based gravity models have been proposed as predictive alternatives to traditional trip distribution formulations, directly estimating OD flows from zonal attributes [31]. Other work has focused on zone-to-zone travel demand forecasting using data-driven models [32], mode choice prediction using machine learning classifiers [33], or approximating traffic assignment and equilibrium behaviour using graph neural networks [34]. These studies demonstrate that machine learning can improve flexibility and predictive performance for specific components of the demand modelling pipeline. However, most existing approaches focus on approximating individual components of the FSM in isolation, are often difficult to interpret, and rarely produce link-level traffic volumes in a fully integrated and scalable manner.

These observations highlight complementary strengths and limitations across modelling paradigms. FSM offers interpretability and theoretical structure but can be limited in flexibility, scalability, and calibration efficiency. Data-driven models offer the potential to improve prediction accuracy by uncovering non-linear relationships and leveraging diverse data sources, but they are often not designed for long-term planning applications and rarely target full segment-level demand estimation. There remains a need for a modelling framework that preserves the strategic planning orientation of the FSM while leveraging modern machine learning to directly estimate segment-level traffic volumes.

In this study, we propose Mukara, a deep learning framework that approximates the aggregate relationship between external demand-related and network features and observed highway traffic volumes. The objectives are threefold: (1) to develop a data-driven model that directly estimates national-scale highway traffic volumes from socioeconomic characteristics and road network structure; (2) to evaluate its predictive performance and spatial generalisability by benchmarking it against baseline models; and (3) to examine the contribution of different feature groups to prediction accuracy through ablation analysis. We name our model “Mukara”, derived from the Japanese term meaning “from nothing”, reflecting its objective of predicting traffic without relying on historical sensor readings.

## Materials and methods

### Overview

We now introduce the workflow of this study. First, we construct a highway trunk road network, where the graph structure encodes connectivity information relevant to trip distribution across OD pairs. Each road segment is enriched with attributes such as driving distance and driving duration, which correspond to the generalised travel cost inputs typically used in the assignment step of FSM. Next, we integrate rasterised geographic datasets including population density, employment statistics, land use areas, the number of various types of POIs, and aggregated measures of local road infrastructure. These inputs serve to approximate key elements of trip generation and mode choice, capturing the spatial and socioeconomic determinants of travel demand that are traditionally modelled using regression and discrete choice models within FSM [11]. They have also been shown to be fundamental determinants of travel demand across the broader transport and urban economics literature, consistently shaping trip frequencies, spatial interaction patterns, and network flows [35–38]. Ground truth traffic volume data is aligned with corresponding road segments to enable supervised learning.

**Fig 1:**
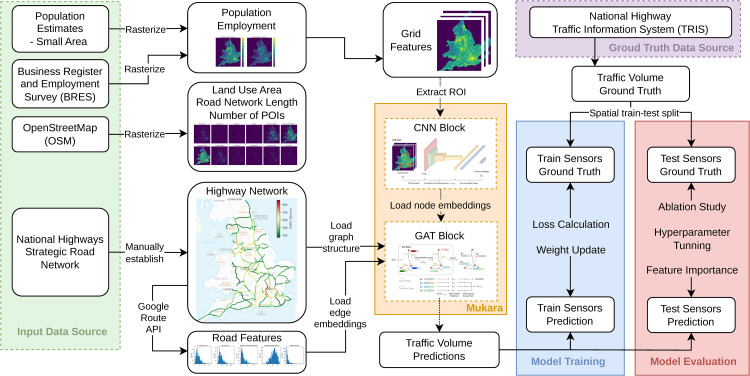
Overview of the Mukara workflow.

Mukara is trained using sensor-labelled road segments in the training set and evaluated on spatially distinct test segments to assess spatial generalisability. By structuring inputs around the core components of FSM and learning end-to-end mappings to observed traffic volumes, Mukara provides a data-driven predictive approximation of the overall demand-to-flow process without explicitly modelling intermediate behavioural stages. Fig 1 illustrates the complete methodological pipeline. Table 2 is a summary of all symbols used in this study. Table 3 provides a summary of all data sources, including the source agency, time coverage, spatial resolution, and intended usage within the study.

**Table 2:** Summary of notations used in the Mukara model.

Notation	Description
𝒢=(𝒱,ℰ)	Directed graph representing the highway trunk road network
$𝒱=\{v_{i}\}$	Set of nodes (junctions) in the graph; $v_{i}$ denotes a specific node
$ℰ=\{e_{ij}\}$	Set of edges (road segments); $e_{ij}$ denotes an edge from $v_{i}$ to $v_{j}$
$N_{v},N_{e},N_{t}$	Number of nodes, edges, and time slices (years), respectively
ℳ	Grid-based input features across all years and all channels
${ℳ}_{t}$	Grid-based input features for year *t*
${ℳ}_{pe}$	Population and employment features across all years
${ℳ}_{lp}$	Land use and POI features across all years (broadcast)
$D_{e}$	Number of edge features
$D_{h},D_{w}$	Height and width of the grid-based inputs
$D_{pe},D_{lp},D_{m}$	Number of channels for population/employment, land use/POI, and their sum respectively
$𝒴=\{y_{t,e_{ij}}\}$	Set of observed (ground truth) traffic volumes for each edge $e_{ij}$ and year *t*
$\hat{𝒴}=\{{\hat{y}}_{t,e_{ij}}\}$	Set of predicted traffic volumes for each edge $e_{ij}$ and year *t*
$𝒳=\{{𝐱}_{e_{ij}}\}$	Set of raw feature vectors for each edge $e_{ij}$
*d*	Dimension of the latent space for both node and edge embeddings
${𝐡}_{v_{i}}^{l}$	Node embedding of $v_{i}$ at GAT layer *l*
${𝐡}_{e_{ij}}$	Edge embedding of $e_{ij}$ after MLP
$L_{\text{CNN}},L_{\text{GAT}}$	Number of layers in the CNN and GAT blocks, respectively
*K*	Number of attention heads in the GAT layer
*k*	Index of a specific attention head
𝒩(i)	Set of neighbouring nodes of node $v_{i}$
${ℒ}_{\text{train}}$	Training loss used to optimise model parameters

**Table 3:** Summary of data sources used in this study.

Name	Usage Category	Spatial Resolution	Time Period	Source
National Highways Strategic Road Network	Highway structure	National roads	2023	National Highways [39]
TRIS Traffic Volume	Ground truth label	Sensor-level	2015–2022	National Highways TRIS [40]
Google Routes API	Road segment attributes	Segment-level	Accessed 2023	Google Routes API
Population Estimates	Population input features	LSOA-level rasterised to 1 km grid	2015–2022	ONS / NOMIS [41]
Employment (BRES)	Employment input features	LSOA-level rasterised to 1 km grid	2015–2022	ONS / NOMIS [42]
LSOA Boundaries	Rasterisation and spatial reference	LSOA-level	2011, 2021	ONS Geography [43,44]
OpenStreetMap (via Geofabrik)	Land use, road attributes, POI data	Element-level rasterised to 1 km grid	Snapshot: 2023-01-01	OSM [45]
UK Country Boundary (ONS)	Map base layer	Country-level	2023	ONS Geography [46]

### Data

#### Highway network.

To study interurban traffic dynamics, a highway network graph covering the entire region of England was constructed based on the National Highways Strategic Road Network [39]. This graph serves as the backbone for information propagation in Mukara. The network consists of 181 nodes ($N_{v}=181$), representing highway trunk road junctions, typically located near major cities, and 498 edges ($N_{e}=498$), corresponding to 249 highway trunk road segments in both directions. Each edge is assigned a sensor from the National Highway Traffic Information System (TRIS) [40], resulting in a total of 498 sensors. Nodes and edges were selected to capture traffic flow along trunk roads connecting major cities and towns, while avoiding bypasses and highway exits. Details of the sensor selection procedure and the representativeness evaluation of the selected sensors are included in Appendix S2-S3 (S1 File).

**Fig 2:**
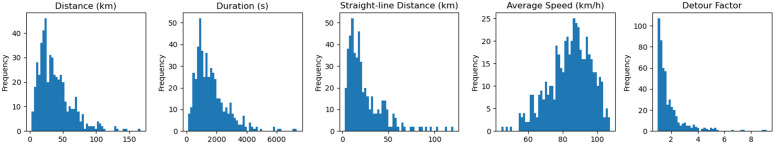
Distributions of the five edge features used in the Mukara model. Namely, driving distance, driving duration, straight-line distance, average driving speed, and detour factor. These metrics were normalised for input into the model.

Features on highway trunk road segments in both directions were collected using the Google Routes Application Programming Interface (API). The extracted edge features include driving duration, driving distance, straight-line distance, average driving speed, and detour factor. Together, these edge features, $𝒳=\{{𝐱}_{e_{ij}}\}$, are structured into a tensor with dimensions $N_{e}\times D_{e}=498\times 5$, where $N_{e}$ is the total number of edges and $D_{e}$ is the number of features. All edge features are normalised before being input into the model. Fig 2 provides a visual summary of the distributions of these edge features.

#### Population and employment.

Population data was sourced from the Population Estimates – Small Area dataset [41], provided by the Office for National Statistics (ONS) through the National Online Manpower Information System (NOMIS) service. This dataset provides annual population estimates for England and Wales at the Lower Layer Super Output Area (LSOA) level, stratified by age group and sex. Employment data was obtained from the Business Register and Employment Survey (BRES) [42], also provided by ONS through NOMIS. This dataset includes employment counts, covering full-time, part-time, and self-employed workers across all industries within England and Wales.

For both datasets, data from the years 2015–2022 were selected. The LSOA-based data was rasterised into a 1 km x 1 km grid using LSOA boundaries provided by ONS [43,44]. This process resulted in a grid tensor ${ℳ}_{pe}$ with dimensions $N_{t}\times D_{h}\times D_{w}\times D_{pe}=8\times 653\times 573\times D_{pe}$, where $N_{t}$ is the number of years, $D_{h}$ and $D_{w}$ are the height and width of the grid, and $D_{pe}$ represents the number of grid channels for population and employment.

**Fig 3:**
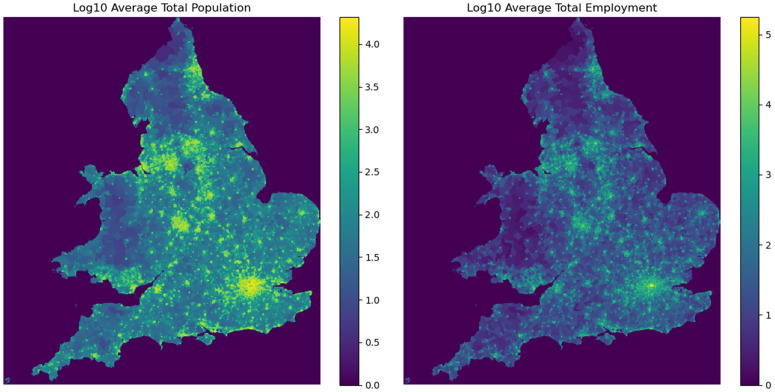
Heat maps of aggregated population and employment across England and Wales in year 2022. Higher intensity indicates areas with larger population and employment density, based on LSOA-level data rasterised to 1 km x 1 km grid.

To account for differences in travel behaviour across demographic groups, separate channels were created for population and employment strata. The input data were stratified accordingly, and Table 4 summarises the resulting input channels. In each training task, all channels or a subset of these channels were selected to analyse the model’s performance under different levels of stratification of the input data. Fig 3 visualises the aggregated population and employment data in 2022 as heat maps.

**Table 4:** Breakdown of raster input channels for population and employment features.

Feature Type	Subcategory	Number of Channels
Population	Total population (aggregated across all demographics)	1
	By sex (male and female)	2
	By age group (five predefined age bands)	5
	By age and sex (age groups further stratified by sex)	10
Employment	Total employment (aggregated across all sectors and types)	1
	By work type (full-time, part-time, self-employed)	3
	By sector (18 industry sectors)	18
	By work type and sector (combinations of the above categories)	54

#### Land use, road network, and POI.

Land use, road network, and POI data were sourced from OpenStreetMap (OSM) and downloaded via Geofabrik’s free download server [45]. The data were extracted from a historical snapshot of the England subregions and Wales .osm.pbf files with the timestamp 2023-01-01. This static snapshot was chosen for all years from 2015 to 2022 to ensure consistency and completeness, as land use and POI data are relatively stable over time. To assess robustness to OSM data vintage, we conducted an additional sensitivity analysis using an alternative historical OSM snapshot (timestamp: 2019-01-01), selected to be temporally closer to the early portion of the study period. Results of this analysis are reported in Appendix S4 (S1 File).

The tags used to extract the data are summarised in Table 5. These include land use classifications (e.g., residential, industrial), road network hierarchies from high-level motorways to low-level residential roads, and a diverse set of POI categories such as transport, food, health, education, and retail facilities. The selection of these tags was based on the Deep Gravity model [31] and their availability in the OSM database.

**Table 5:** The categories, OpenStreetMap (OSM) keys, and associated values used to extract thematic raster layers for input features.

Category	Channel	OSM Key	OSM Values
Land Use	Residential	landuse	residential
	Commercial	landuse	commercial
	Industrial	landuse	industrial
	Retail	landuse	retail
Road Network	High Level	highway	motorway, trunk, primary
	Medium Level	highway	secondary, tertiary
	Low Level	highway	residential, unclassified
POI	Transport	amenity	bus_station, parking
		railway	station, stop, tram_stop
	Food	amenity	bar, cafe, restaurant
	Health	amenity	clinic, hospital, pharmacy
	Education	amenity	school, college, kindergarten, university
	Retail	shop	supermarket, department_store, mall

**Fig 4:**
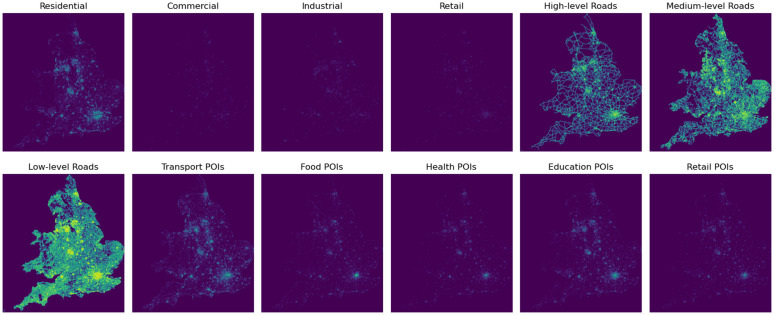
Heat maps showing the distribution of 12 features including land use, road network, and POI across England and Wales. The features were aggregated to 1 km x 1 km grid.

For each grid cell, the following metrics were calculated: total area of each land use type, total length of roads for each hierarchical level, and total number of POIs for each category. These metrics were then aggregated into a grid tensor, ${M^{\prime }}_{lp}$, with dimensions $D_{h}\times D_{w}\times D_{lp}=653\times 573\times 12$, where $D_{h}$ and $D_{w}$ represent the height and width of the grid, and $D_{lp}$ is the number of grid channels corresponding to the land use and POI categories. Fig 4 visualises these features as heat maps. To construct the final grid features ℳ, the tensor ${M^{\prime }}_{lp}$ was broadcast on the time axis resulting in ${ℳ}_{lp}$, which was then concatenated with the population and employment tensor ${ℳ}_{pe}$ along the channel dimension: $ℳ={ℳ}_{pe}\|{ℳ}_{lp}$.

#### Traffic volume.

The ground truth traffic volume data were sourced from the Traffic Information System (TRIS), managed by National Highways [47]. TRIS provides comprehensive data on traffic speed and volume, collected in 15-minute intervals using loop sensors. Across England, 19,364 sensors are integrated into this network, which has been operational since 2014. The data used in this study were accessed and downloaded through the API provided by TRIS [40].

**Fig 5:**
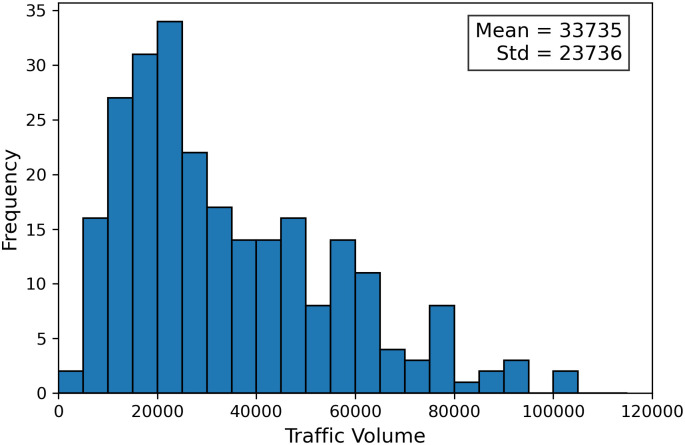
Histogram of average weekday daily traffic volumes. The histogram shows traffic volumes across the 498 sensors, calculated over 8 years (2015–2022).

**Fig 6:**
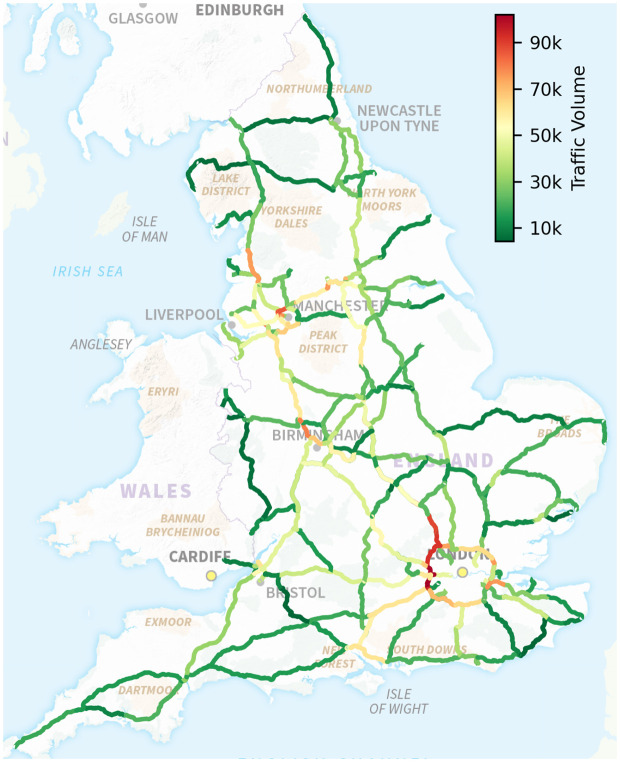
Spatial visualisation of average weekday daily traffic volumes. The figure shows traffic levels across the 498 sensors, averaged over 8 years (2015–2022) and aggregated for both directions of the same highway segment.

To align the traffic volume data with the input features, we used 8 years of traffic volume records from 1 January 2015 to 31 December 2022, for each of the 498 sensors included in the established highway network. The mean weekday daily traffic volume was calculated for each year and each sensor to generate the ground truth tensor $𝒴=\{y_{t,e_{ij}}\}$, with dimensions $N_{t}\times N_{e}=8\times 498$. Weekends and bank holidays are excluded to focus on regular traffic patterns. A sensitivity analysis of including weekends and holidays is provided in Appendix S5 (S1 File). This mean-based aggregation serves to smooth out daily variability and reduce noise caused by anomalous or irregular traffic days. The resulting average reflects the typical structural demand for highway usage under normal conditions, aligning with the planning-level nature of our study. Fig 5 shows a histogram of the average traffic volumes across the 498 sensors over the 8-year period, illustrating the variability in traffic levels. Fig 6 provides a spatial visualisation of these volumes, averaged over both directions.

### Mukara model

The goal of the Mukara model is to predict weekday daily traffic volumes for all highway segments in a given year, using only external features. The model leverages three primary inputs: the graph structure of the highway network, edge-level characteristics, and grid-based contextual features for the corresponding year. The prediction task for year *t* is formally defined as:


$${\hat{𝒴}}_{t}=\text{Mukara}(𝒢,𝒳,{ℳ}_{t})$$ (1)

where ${\hat{𝒴}}_{t}\in {\mathbb{R}}^{N_{e}}$ denotes the set of predicted traffic volumes for all edges in year *t*; 𝒢=(𝒱,ℰ) represents the highway network graph, where 𝒱 is *t*he set of nodes and ℰ is the set of directed edges; $𝒳=\{{𝐱}_{e_{ij}}|e_{ij}\in ℰ\}$ is the set of raw feature vectors for each edge $e_{ij}$; and ${ℳ}_{t}\in {\mathbb{R}}^{D_{h}\times D_{w}\times D_{m}}$ is the rasterised grid-based input tensor for year *t*. For notational simplicity, we omit the time subscript *t* in the remainder of this section where the context is clear.

**Fig 7:**
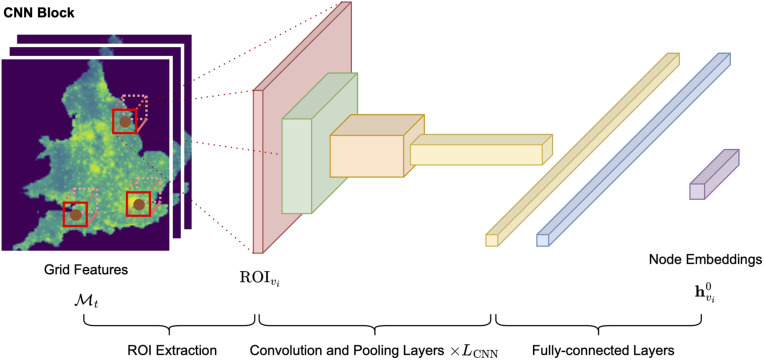
A visual representation of the CNN block in the Mukara model. The block processes grid features (${ℳ}_{t}$) by extracting regions of interest (ROIs) around each node, applying convolutional and pooling layers, and generating node embeddings (${𝐡}_{v_{i}}^{0}$) for subsequent graph attention processing.

**Fig 8:**
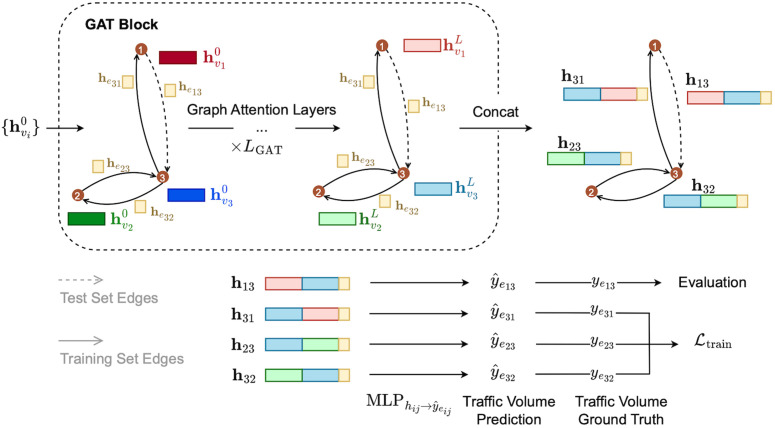
An overview of the GAT block in the Mukara model. Initial node embeddings (${𝐡}_{v_{i}}^{0}$) are refined through multiple GAT layers, incorporating edge embeddings (${𝐡}_{e_{ij}}$). The final embeddings are concatenated and passed through an MLP to predict traffic volumes for edges. Solid and dashed lines represent training and test edges, respectively.

To efficiently pass information from inputs to outputs, the Mukara model is composed of two main building blocks: a CNN block for processing spatial grid features, and a GAT block for capturing topological and relational information from the network. An overview of these blocks is provided in Figs 7 and 8.

The CNN block is responsible for processing the grid features ${ℳ}_{t}$ to generate node-specific embeddings by extracting and encoding information from a fixed-size Region of Interest (ROI) centred around each node. CNNs are particularly suitable for this task, as they not only flatten the grid into a usable vector but also extract rich spatial patterns (e.g., density gradients, clustering effects) that are often lost in simple aggregations. This enables more nuanced representation of local environmental context. For each node $v_{i}$, its geographic coordinates are used to locate the corresponding centre pixel in ${ℳ}_{t}$. A square ROI of fixed size that is aligned with the pixel grid and centred at this location is extracted to represent the spatial context around the node. If the ROI extends beyond the boundaries of the grid, zero-padding is applied to maintain consistent input dimensions. The ROI size, defined in pixel units, corresponds to a real-world spatial area (e.g., 25 km × 25 km) and is treated as a tunable hyperparameter. The extracted ROIs are also three-dimensional tensors, with the first two dimensions representing the spatial extent (height and width), and the third dimension representing the number of feature channels in ${ℳ}_{t}$.

Each ROI is passed through a series of convolutional layers with depth *L*_CNN_, followed by Rectified Linear Unit (ReLU) activations and max-pooling operations. The final convolutional output is flattened into a one-dimensional vector, producing the initial node embedding ${𝐡}_{v_{i}}^{0}$. The entire CNN transformation can be expressed as:


$${𝐡}_{v_{i}}^{0}=\text{Flatten}\left({\left(\text{Pooling}\circ \text{ReLU}\circ \text{Conv}\right)}^{L_{\text{CNN}}}\left({\text{ROI}}_{v_{i}}\right)\right),\;\text{where}\;{\text{ROI}}_{v_{i}}\subset {ℳ}_{t}$$ (2)

where ${𝐡}_{v_{i}}^{0}\in {\mathbb{R}}^{d}$ is the initial embedding of node $v_{i}$ produced by the CNN block; *L*_CNN_ is the number of convolutional layers, ∘ denotes function composition; and ${\text{ROI}}_{v_{i}}$ is the region of interest centred at node $v_{i}$ extracted from the grid input ${ℳ}_{t}$.

This initial node embedding captures the spatial and socioeconomic context surrounding each node, which is related to the trip generation process, and serves as the starting point for graph-based reasoning.

Following the CNN block, the GAT block refines the node embeddings by integrating information from neighbouring nodes and the attributes of the connecting edges. Each edge embedding ${𝐡}_{e_{ij}}$ is generated by applying a Multi-Layer Perceptron (MLP) to the raw edge feature vector ${𝐱}_{e_{ij}}$:


$${𝐡}_{e_{ij}}={\text{MLP}}_{\text{edge}}({𝐱}_{e_{ij}})$$ (3)

where ${𝐱}_{e_{ij}}\in {\mathbb{R}}^{D_{e}}$ is the raw feature vector associated with edge $e_{ij}$, and ${\text{MLP}}_{\text{edge}}(\cdot )$ denotes the shared edge embedding layer applied across all edges. The output of this layer is the edge embedding ${𝐡}_{e_{ij}}\in {\mathbb{R}}^{d}$.

The GAT block operates over multiple layers, each performing an attention-based message passing step. At each layer *l*, the node embeddings are updated by attending to their neighbours and the features of the connecting edges, based on the graph structure:


$${𝐡}_{v_{i}}^{l}={\text{GAT}}^{l}({𝐡}_{v_{i}}^{l-1},{𝐡}_{e_{ij}},𝒢)\;\text{for}\;l\in \{1,2,\ldots ,L_{\text{GAT}}\}$$ (4)

where ${𝐡}_{v_{i}}^{l}\in {\mathbb{R}}^{d}$ is the embedding of node $v_{i}$ at layer *l*; ${𝐡}_{v_{i}}^{l-1}$ is the embedding from the previous layer; ${𝐡}_{e_{ij}}$ is the embedding of the edge connecting $v_{i}$ and its neighbour $v_{j}$; and 𝒢=(𝒱,ℰ) is the input graph. The function ${\text{GAT}}^{l}(\cdot )$ represents the graph attention operation of the *l*-th GAT layer.

The attention mechanism computes unnormalised scores $\beta_{ij}$ that quantify the importance of node $v_{j}$ to node $v_{i}$, based on their respective embeddings and the edge connecting them:


$$\beta_{ij}=\text{LeakyReLU}\left({𝐚}^{⊤}\left[{𝐖}_{v}{𝐡}_{v_{i}}^{l-1}\|{𝐖}_{v}{𝐡}_{v_{j}}^{l-1}\|{𝐖}_{e}{𝐡}_{e_{ij}}\right]\right)$$ (5)

where ${𝐖}_{v}$ and ${𝐖}_{e}$ are learnable linear projection matrices applied to node and edge embeddings, respectively; ${𝐚}^{⊤}$ is a learnable attention vector; and ‖ denotes vector concatenation. To explicitly incorporate edge information, the edge embedding ${𝐡}_{e_{ij}}$ is included in the attention mechanism. This allows the model to modulate attention weights not only based on node content but also on attributes of the edge (e.g., distance, speed, or detour factor), which are highly relevant for traffic flow. As a result, the edge-aware attention improves the ability of the model to capture meaningful spatial interactions in the highway network. For clarity, we omit the attention head index *k* in the notation for $\beta_{ij}$ and $\alpha_{ij}$, although in practice, separate sets of attention parameters are learned for each of the *K* attention heads at each layer.

Within each layer, the attention coefficients $\alpha_{ij}$ are computed by normalising the attention scores $\beta_{ij}$ across the neighbouring nodes 𝒩(i) of node $v_{i}$:


$$\alpha_{ij}=\frac{\exp(\beta_{ij})}{\sum_{m\in 𝒩(i)}\exp(\beta_{im})}$$ (6)

Using these attention coefficients, each node updates its embedding for attention head *k* by aggregating the transformed embeddings of its neighbours:


$${𝐡}_{v_{i},k}^{l}=\sigma \left(\sum_{j\in 𝒩(i)}\alpha_{ij}{𝐖}_{v}{𝐡}_{v_{j}}^{l-1}\right)$$ (7)

where σ(·) denotes a non-linear activation function such as ReLU, and k∈{1,…,K} indexes different attention heads.

The outputs from all *K* attention heads are concatenated and passed through a MLP to produce the updated embedding for node $v_{i}$ at layer *l*:


$${𝐡}_{v_{i}}^{l}={\text{MLP}}_{\text{agg}}^{l}\left(\text{Concat}\left({𝐡}_{v_{i},1}^{l},{𝐡}_{v_{i},2}^{l},\ldots ,{𝐡}_{v_{i},K}^{l}\right)\right)$$ (8)

The GAT block sequentially updates node embeddings by calculating attention scores, normalising these scores, and aggregating neighbour information across *L*_GAT_ layers. By the end of the process, the node embeddings encapsulate both local contexts and wider network information.

Finally, traffic volume predictions for each edge are obtained by concatenating the final embeddings of the origin node (${𝐡}_{v_{i}}^{L}$), the destination node (${𝐡}_{v_{j}}^{L}$), and the edge embedding (${𝐡}_{e_{ij}}$), and passing the result through a prediction MLP:


$${\hat{y}}_{e_{ij}}={\text{MLP}}_{\text{pred}}\left({𝐡}_{v_{i}}^{L}\|{𝐡}_{v_{j}}^{L}\|{𝐡}_{e_{ij}}\right)$$ (9)

This structured design enables Mukara to effectively capture spatial, relational, and feature-based dependencies, leading to accurate predictions of traffic volumes across the highway network.

### Model training and experimental settings

#### Loss function and evaluation metrics.

The Mukara model is trained using the mean of the Geoffrey E. Havers (GEH) statistic [48]—hereafter referred to as MGEH—a metric widely used to evaluate the goodness-of-fit of traffic models. The GEH statistic accounts for both the absolute difference and the percentage difference between the modelled and observed flows, making it particularly suitable for traffic volume prediction tasks. Unlike the commonly used Mean Squared Error (MSE), GEH emphasises proportionality, allowing errors to be evaluated relative to the magnitude of the observed volumes. A recent study has shown that the GEH loss function is consistent and outperforms Mean Absolute Error (MAE) and MSE in most cases [49].

The GEH statistic for an individual edge $e_{ij}$ is defined as:


$${\text{GEH}}_{e_{ij}}=\sqrt{2\cdot \frac{(y_{e_{ij}}-{\hat{y}}_{e_{ij}}{)}^{2}}{y_{e_{ij}}+{\hat{y}}_{e_{ij}}}}$$ (10)

where $y_{e_{ij}}$ is the observed traffic volume and ${\hat{y}}_{e_{ij}}$ is the predicted traffic volume for edge $e_{ij}$. The time subscript *t* in this equation and the equations for MAE and MSE are omitted for simplicity.

For evaluation, in addition to MGEH, we report the Mean Absolute Error (MAE) and the coefficient of determination *R*^2^. MAE, MSE, and the coefficient of determination *R*^2^ are defined as follows:


$$\text{MAE}=\frac{1}{N_{e}}\sum_{e_{ij}\in ℰ}\left|y_{e_{ij}}-{\hat{y}}_{e_{ij}}\right|$$ (11)

$$\text{MSE}=\frac{1}{N_{e}}\sum_{e_{ij}\in ℰ}{\left(y_{e_{ij}}-{\hat{y}}_{e_{ij}}\right)}^{2}$$ (12)

$$R^{2}=1-\frac{\sum_{e_{ij}\in ℰ}{\left(y_{e_{ij}}-{\hat{y}}_{e_{ij}}\right)}^{2}}{\sum_{e_{ij}\in ℰ}{\left(y_{e_{ij}}-\bar{y}\right)}^{2}}$$ (13)

where $\bar{y}$ denotes the mean observed traffic volume across all evaluated edges.

The MGEH loss function used for training is the mean of the GEH statistics across all edges:


$${ℒ}_{\text{train}}=\text{MGEH}=\frac{1}{N_{e}}\sum_{e_{ij}\in ℰ}{\text{GEH}}_{e_{ij}}$$ (14)

To assess the robustness of this choice of objective function, we conducted a loss-function sensitivity analysis (Appendix S6, S1 File), in which Mukara was re-trained using alternative objectives (MSE, MAE, and Huber loss) under the same spatially blocked cross-validation protocol. The results indicate that aggregate predictive performance remains broadly consistent across loss specifications.

#### Training algorithm.

Model training and evaluation were conducted using both random cross-validation (CV) and spatial CV. For CV, a five-fold scheme was adopted. In each iteration, one fold was held out as the test set, while the remaining four folds were used for model training. This process was repeated five times so that each subset served as the test set once. For spatial CV, the 498 highway trunk-road segments were grouped according to the nine official regions of England. A nine-fold spatial CV procedure was then implemented, in which all segments within one region were held out as the test set in each fold, while the remaining eight regions constituted the training set. Ground truths for segments in the test region were used exclusively for evaluation and were not accessed during model training. This evaluation design is consistent with the study’s objective of assessing the feasibility of predicting traffic volumes for geographically unobserved highway segments, thereby providing a stringent test of spatial transferability.

As shown in Algorithm 1, the training process involves iteratively selecting one year of grid features from the training data, performing a forward pass to make predictions, calculating the loss to measure prediction errors, and conducting a backward pass to compute gradients. The parameters are updated after each batch, and this cycle is repeated for a predefined number of epochs. While using the entire year of training samples as a batch is quite computationally generous, it ensures that the model learns from all sensors simultaneously. Experiments showed that this approach achieves lower loss compared to splitting the samples into smaller batches.

**Algorithm 1 Training algorithm for the Mukara model**

**Input:** Highway network graph 𝒢=(𝒱,ℰ), edge features $𝒳=\{{𝐱}_{e_{ij}}\}$, grid features $\{{ℳ}_{t}{\}}_{t=1}^{N_{t}}$.

**Output:** Predicted traffic volumes $\{{𝒴}_{t}{\}}_{t=1}^{N_{t}}$.

1:   Split sensors into five folds, and select one fold as the test set.

2:   Initialise model parameters θ.

3:   **for** epoch = 1 to *N*_epochs_ **do**

4:     **for** each year *t* in training data **do**

5:       Extract grid features ${ℳ}_{t}$ from ℳ.

6:       Extract ground truth traffic volumes ${𝒴}_{t}$ from 𝒴 for all sensors.

7:       Perform a forward pass through the Mukara model:


$$\hat{{𝒴}_{t}}=\text{Mukara}(𝒢,𝒳,{ℳ}_{t}).$$

8:       Compute the training loss:


$${ℒ}_{\text{train}}=\frac{1}{N_{e}}\sum_{e_{ij}\in ℰ}\sqrt{2\cdot \frac{(y_{e_{ij}}-{\hat{y}}_{e_{ij}}{)}^{2}}{y_{e_{ij}}+{\hat{y}}_{e_{ij}}}}.$$

9:       Compute gradients: $\nabla_{\theta }{ℒ}_{\text{train}}$.

10:       Update parameters using gradient descent with learning rate α:


$$\theta \leftarrow \theta -\alpha \nabla_{\theta }{ℒ}_{\text{train}}.$$

11:   **end for**

12: **end for**

13: **Return:** Trained Mukara model and predicted traffic volumes $\hat{{𝒴}_{t}}$.

#### Experimental settings.

For the default grid features, we use aggregated population, aggregated employment, all land use, road network, and POI features, resulting in a total of $D_{pe}=14$ channels for the grid tensor. The default model hyperparameters are as follows: The ROI size is set to 25, corresponding to 25 km, which covers typical spatial extents of small to medium-sized UK cities and aligns with observed urban activity ranges such as commuting distances and economic catchment areas. This choice ensures that the model captures sufficient spatial context without introducing excessive noise from distant, unrelated regions. The CNN block consists of *L*_CNN_ = 3 layers with channel sizes of 16, 32, and 64 for each layer. The kernel size is set to 3, strides are set to 1, and max pooling is applied with a pool size of 2 and strides of 2, effectively reducing spatial dimensions while preserving relevant feature patterns. The output dense layer of the CNN block, which also serves as the node embedding size in the GAT block, is set to 16 to balance representational capacity and computational efficiency. The GAT block is composed of *L*_GAT_ = 5 layers, each employing 3 attention heads to capture diverse relational patterns among neighbouring nodes and edges. All MLPs used in the model have a hidden size of 16 with ReLU as the activation function and an output size of 16. Each batch corresponds to one year of data; therefore, there are 8 batches in one epoch.

Training is performed using the Adam optimiser with a learning rate of 0.001 and gradient clipping at 5 to ensure stability. The experiments were conducted on a system equipped with an Intel i7 CPU, 16 GB of RAM, and a single NVIDIA RTX 4060 Ti GPU. The software environment included Windows 11 as the operating system, Python 3.9.18, TensorFlow 2.10.1, and Deep Graph Library (DGL) version 1.1.2 with CUDA 11.8 support.

Following best practices in empirical forecasting and applied machine learning [50–53], we introduce three commensurate benchmark models evaluated under identical CV protocols and performance metrics: (1) Ridge regression (L2 regularised linear regression) using the same segment-level feature set as Mukara; (2) a gravity-interaction baseline, a classical distance-decay formulation based on aggregated population and employment “masses” linked to observed traffic volumes via log-linear regression; (3) Random forest regressor, a non-linear ensemble model trained using the identical feature set. All baseline models are trained and evaluated under both random CV and spatial CV. Hyperparameters are tuned strictly within training folds to prevent leakage. Full methodological details for these baselines are provided in Appendix S7 (S1 File).

## Results

### Ablation study and tuning

In the first experiment, we conducted a grid search to identify optimal settings for the Mukara model. As shown in Table 6, the tuned hyperparameters included the number of channels in each CNN layer, the ROI size, the depth of the GAT block *L*_GAT_, the number of attention heads, and the dimensions of the node embeddings. Each model was trained for a maximum of 50 epochs, and the lowest MGEH and MAE losses were recorded.

**Table 6:** Model variants included in the ablation study.

Model Variant	Description
Edge-only model	Only edge features are used; no node embeddings or grid features are included.
No GAT layers	Uses OD node and edge features, but disables GAT layers (*L*_GAT_ = 0).
GAT depth	Number of GAT layers varied from 1 to 8 to test how many hops of neighbourhood information improve predictions.
CNN channels	CNN channel widths tested: [8,16,32], [16,32,64], [32,64,128], to evaluate CNN capacity.
ROI size	Region-of-interest sizes tested: 21×21, 25×25, 31×31, 41×41 pixels, to extract spatial features around each node.
Number of attention heads	GAT head counts varied from 1 to 5 to assess benefits of multi-head attention.
Node embedding size	Embedding dimensions tested: 8, 16, 32, 64, 128, to explore effect of representation size.

**Fig 9:**
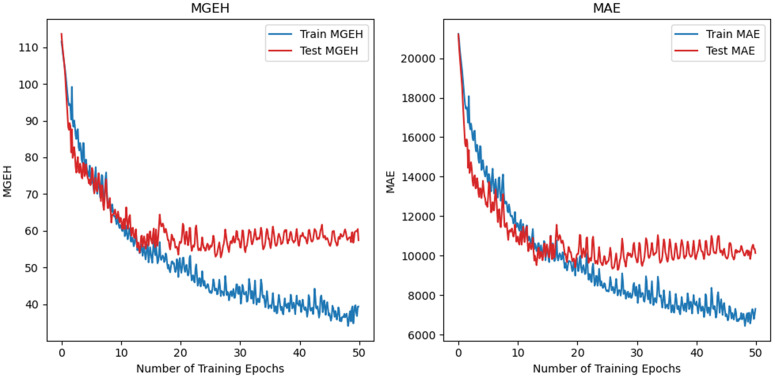
Learning curve of the default Mukara model.

The learning curve for the default model is shown in Fig 9. The curve demonstrates that the model learns effectively, with the lowest loss occurring around the 27th epoch. After this point, the model begins to overfit, as indicated by a gradual increase in test loss. Other models also tend to reach their best performance around this point, suggesting that 50 epochs are sufficient for the learning task. An early stopping mechanism was also examined, with a patience of 10 epochs and a learning rate decay schedule starting from 0.01 and decaying by a factor of 10 down to 0.00001. The best performance achieved under this new setting matches the peak performance without the mechanism.

**Fig 10:**
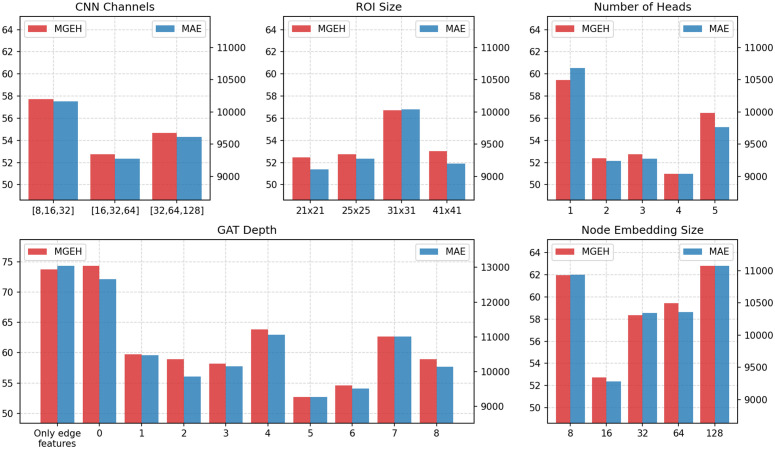
Loss values (MGEH and MAE) in the test set for models with different hyperparameter configurations.

The results of the hyperparameter tuning are presented in Fig 10. The optimal settings were found to be CNN channels of [16, 32, 64], an ROI size of 21 km x 21 km, 4 attention heads, a GAT depth (*L*_GAT_) of 5, and a node embedding size of 16. Based on these findings, the default model was updated to include 4 attention heads while retaining the other hyperparameter settings.

Several observations can be drawn from these experiments. First, the simplest model, which relies solely on edge features for prediction and does not use grid features or node embeddings, results in high loss. This finding emphasises that geographic and contextual information captured in the node embeddings is essential, as edge embeddings alone are insufficient for accurate predictions. A slightly more complex model that incorporates OD node embeddings in addition to edge features, but excludes GAT layers (*L*_GAT_ = 0), also yields high loss. This demonstrates that adding only origin and destination embeddings to the edge representation is not sufficient for effective predictions. Models with a GAT depth of 5 or 6 achieved the lowest losses, suggesting that incorporating information from nodes up to 5 or 6 degrees away significantly enhances the model’s predictive capability. However, increasing the depth beyond this point led to overfitting.

The inclusion of multiple attention heads also improved performance, highlighting the benefit of passing multiple channels of information through the network. This effect is analogous to increasing the number of feature maps in CNNs, enhancing the model’s ability to capture diverse patterns and relationships.

Finally, the dimensions of the CNN channels and node embeddings were most effective when balanced. Channels and embeddings that were too small resulted in underfitting, as the model failed to capture sufficient information. Conversely, excessively large dimensions led to overfitting, where the model struggled to generalise due to capturing irrelevant or noisy features.

### Performance evaluation

**Fig 11:**
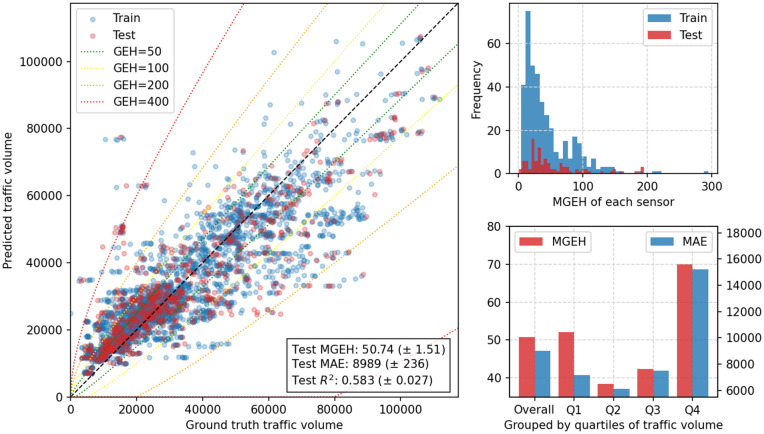
Prediction performance of the Mukara model. (Left) Scatter plot comparing predicted traffic volumes with ground truth values for all sensor-year points, with GEH boundaries for reference. (Upper right) Histogram of mean GEH for each sensor, averaged over 8 years. (Lower right) Bar plots of MGEH and MAE for sensors grouped by traffic volume quartiles. Results are for the first fold of the cross-validation. Metrics shown are mean and standard deviation across folds.

**Fig 12:**
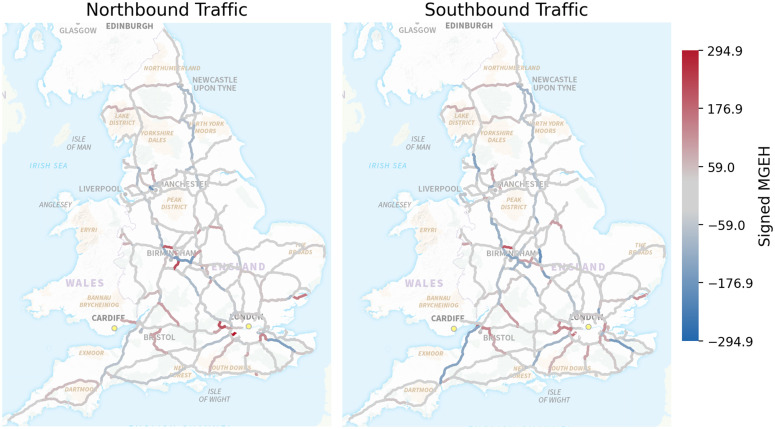
Error maps showing signed MGEH values for northbound and southbound traffic. Positive values (red) indicate overestimation, while negative values (blue) indicate underestimation. The maps reveal localised errors, particularly around areas such as Manchester, but no clear geographical trends overall.

We evaluated the Mukara model using the optimal configuration identified through hyperparameter tuning. For each cross-validation scheme, the model was retrained within the training folds and evaluated exclusively on held-out data. The configuration achieving the lowest MGEH within the validation procedure was retained. The trained models were then used to generate traffic volume estimates for all eight years across the 498 highway trunk-road sensors. The corresponding results are presented in Figs 11 and 12.

Table 7 summarises comparative performance across models and validation schemes. Under random 5-fold CV, Mukara achieves a mean test MGEH of 50.74 (1.51), a test MAE of 8,989 (236) vehicles per day, and an *R*^2^ of 0.583 (0.027). These results substantially outperform all baseline models. The gravity model yields an MGEH of 84.23 (2.47) and an MAE of 14,836 (419), while ridge regression and random forest reduce errors further but remain clearly inferior to Mukara. The global mean predictor performs worst, as expected.

**Table 7:** Model performance under random 5-fold CV and spatially blocked CV. Values are reported as mean (standard deviation) across folds.

Model	MGEH	MAE	$R^{2}$
**Random 5-fold CV**			
Global Mean Predictor	102.31 (1.84)	19708 (313)	0
Classical Gravity Model	84.23 (2.47)	14836 (419)	0.342 (0.021)
Ridge Regression (L2)	75.64 (1.98)	12942 (365)	0.463 (0.024)
Random Forest Regressor	71.38 (1.76)	12319 (341)	0.504 (0.022)
Mukara (GAT-based)	50.74 (1.51)	8989 (236)	0.583 (0.027)
**Spatially Blocked CV**			
Global Mean Predictor	105.27 (4.63)	20413 (812)	< 0
Classical Gravity Model	93.18 (6.14)	16925 (1042)	0.201 (0.053)
Ridge Regression (L2)	86.72 (5.48)	15109 (928)	0.302 (0.061)
Random Forest Regressor	81.54 (5.22)	14216 (874)	0.361 (0.057)
Mukara (GAT-based)	57.63 (3.42)	9955 (612)	0.521 (0.072)

Under spatial CV, overall performance decreases modestly for all models, reflecting the more stringent evaluation setting. Mukara attains a mean MGEH of 57.63 (3.42), an MAE of 9,955 (612), and an *R*^2^ of 0.521 (0.072). Importantly, the relative performance ranking remains unchanged, and no systematic degradation is observed across regions. Slightly higher errors are observed in folds corresponding to London and the South West, which likely reflect distinct traffic regimes (extremely high-volume urban segments and lower-volume rural segments, respectively). The consistent advantage of Mukara under spatial CV demonstrates that the model generalises to geographically unseen regions. The performance of ridge regression and random forest under spatial CV is comparable to that of the Mukara variant with GAT depth of 0, indicating that models relying solely on local link-level and endpoint features achieve similar predictive capacity. The additional gains observed in the full Mukara configuration therefore arise from multi-hop message passing and structural context propagation across the road network. This confirms that incorporating non-local relational information provides measurable benefits over purely local models.

Fig 11 illustrates detailed prediction performance. The left panel presents a scatter plot comparing predicted traffic volumes with grounds across all sensor–year observations, with GEH reference thresholds overlaid. The upper-right panel shows the distribution of mean GEH values for each sensor averaged over eight years. The lower-right panel reports MGEH and MAE grouped by traffic volume quartiles. Consistent with Table 7, errors are larger for sensors with extremely low or extremely high traffic volumes, suggesting that extreme traffic regimes remain more challenging to model than medium-range volumes.

GEH reference ranges are commonly used as diagnostic guidelines rather than formal acceptance criteria. Because the GEH statistic scales with flow magnitude, higher reference ranges are typically applied when evaluating daily mean traffic volumes compared to hourly counts. Following established practice in large-scale assignment validation [48,54], values below 16 are treated as indicative of close agreement and values between 16 and 32 as reflecting moderate deviation for daily volumes. In the test sets under random CV, 18% of sensors achieve a MGEH below 16, and 49% fall below 32. The empirical distribution of MGEH across sensors further indicates a right-skewed pattern, with the 25th, 50th (median), and 75th percentiles equal to 23.88, 32.10, and 72.51, respectively.

Fig 12 presents spatial error maps showing signed MGEH values averaged over eight years. Positive values indicate overestimation and negative values indicate underestimation, with separate panels for northbound and southbound traffic. No strong large-scale geographic bias is observed. Errors appear localised rather than regionally systematic, further supporting the model’s spatial robustness.

In addition, we also provide detailed hierarchical aggregation results in Appendix S8 (S1 File), including region-level and national-level observed versus predicted totals under both random and spatial cross-validation. These supplementary tables report absolute and percentage deviations for each region, offering a complementary planning-scale evaluation of aggregation coherence beyond edge-level metrics.

### Feature importance

In this section, we explore the relative importance of various input features in the Mukara model. First, we analyse how different levels of stratification in population and employment affect the model’s performance. As detailed in the population and employment subsection, level 1 stratification includes 7 channels for population (2 for sex and 5 for age) and 21 channels for employment (3 for work type and 18 for sector). Level 2 stratification expands to 10 channels for population and 54 channels for employment.

**Fig 13:**
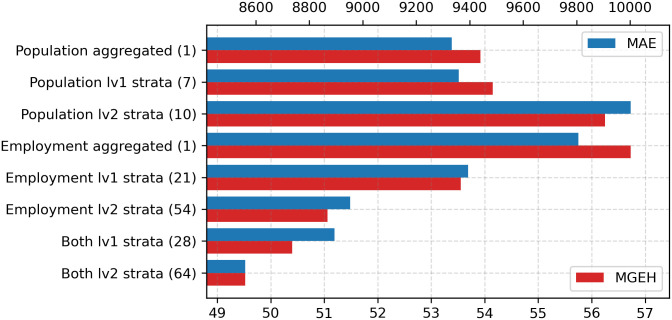
Test set loss values for different population and employment stratification levels. Increased stratification improves model performance for employment and combined features.

The results are illustrated in Fig 13. When population is the sole grid feature, increasing the level of stratification does not significantly reduce the loss. However, for employment, the introduction of stratified channels leads to a marked decrease in loss, particularly for level 2 stratification. Furthermore, when both population and employment are included, the model achieves its lowest loss values with higher stratification levels, surpassing the performance of either feature alone. This indicates that stratification allows the model to capture nuanced patterns in the grid features and leverage interactions between demographic and employment strata, such as age, sex, part-time/full-time employment, and sectors.

**Fig 14:**
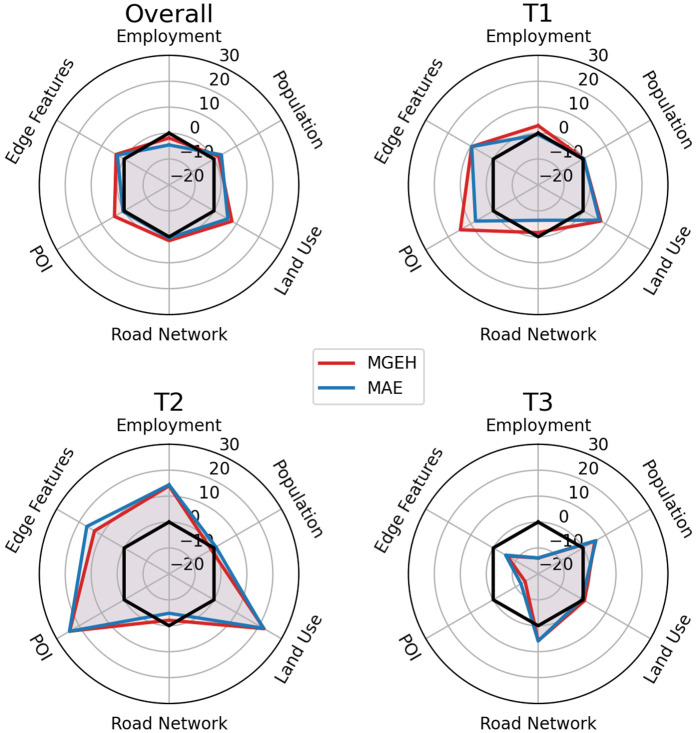
Radar plots showing the percentage change in MGEH (red) and MAE (blue) when individual feature sets are removed. The analysis is presented for overall performance and traffic volume tertiles (low, medium, high). Negative changes indicate a reduction in loss, suggesting possible overfitting or redundancy.

Next, we conduct a feature ablation study to evaluate the importance of each feature set. The full model, which uses all features, serves as the baseline. Six additional models are tested, each omitting one of the following features: population, employment, land use, road network, POI, and edge features. The percentage change in MGEH and MAE loss values is calculated relative to the baseline, revealing the importance of each feature. Fig 14 presents the radar plots summarising these changes across sensors grouped by overall performance and traffic volume tertiles (low, medium, and high levels).

The results show that the removal of any feature generally increases the loss, highlighting their contribution to the model. Notably, land use emerges as the most critical feature, with its removal leading to the largest loss increase across all tertiles. Interestingly, removing employment results in a slight decrease in loss, suggesting possible redundancy or correlation with other features. For sensors with low and medium traffic volumes, employment, land use, POI, and edge features are particularly important, whereas high-traffic sensors exhibit less sensitivity to these features. In fact, for high-volume sensors, the loss reduction upon feature removal suggests potential overfitting or misleading patterns in the training data that fail to generalise to the test set.

These findings underscore the importance of carefully selecting and incorporating features in the Mukara model, as well as the need to account for variations in their relevance across different traffic volume levels. The results also highlight the value of stratifying features to improve the model’s ability to capture complex interactions in the data.

## Discussion

This study proposes a methodological shift in traffic volume prediction by modelling weekday daily highway traffic volumes using an end-to-end deep learning framework that relies exclusively on external socioeconomic and spatial inputs obtainable from official statistics and OSM, without using historical traffic series as model inputs. Using the UK strategic road network as a case study, Mukara achieves a mean test MAE of 8,989 vehicles per day against an average daily traffic volume of 33,734.9 vehicles (relative error ~26.6%) and a mean test *R*^2^ of 0.583 under random 5-fold CV. Under a more stringent nine-fold spatially blocked CV scheme based on England’s official regions, performance remains stable, with an MAE of 9,955 vehicles per day and an *R*^2^ of 0.521. Mukara outperforms all baseline models in both CV settings. The modest reduction in accuracy under geographic hold-out suggests that the model generalises effectively to spatially unseen regions. In comparison, a traditional FSM evaluation on an Istanbul case study reported a best-case %RMSE of approximately 100.92% [14]. Related work also reports lower or comparable performance under different settings and data sources: Das and Tsapakis [55] reported a mean *R*^2^ of 0.36 when predicting annual average daily traffic on low-volume roads using census and survey data, while Ganji et al. [56] achieved *R*^2^ = 0.58 using aerial imagery for urban roads. Narayanan et al. [57] reported higher *R*^2^ values in a metropolitan case study, but relied on synthetic traffic data rather than real-world observations, which typically contain greater noise and heterogeneity.

Importantly, Mukara consistently outperforms all commensurate baseline models evaluated under identical data splits and metrics. Under random CV, the gravity-interaction baseline achieves an *R*^2^ of 0.342, ridge regression 0.463, and random forest 0.504, all substantially below Mukara’s 0.583. Under spatial cross-validation, performance gaps widen further: the gravity model attains an *R*^2^ of 0.201, ridge regression 0.302, and random forest 0.361, compared to Mukara’s 0.521. Similar trends are observed for MAE and MGEH. The baseline models exhibit larger variance and stronger degradation under spatial blocking, indicating greater sensitivity to geographic distribution shifts. These results demonstrate that models relying solely on local link-level and endpoint features—or simple distance-decay formulations—have limited capacity to generalise across regions. By contrast, Mukara’s graph attention architecture with multiple depths captures structural context beyond immediate nodes, enabling information propagation across the network and yielding measurable performance gains under both random and strictly spatial evaluation settings.

From an applied-econometrics perspective [50,51,53], Mukara is framed explicitly as a predictive demand-approximation tool rather than a structural causal estimator. All performance claims are restricted to out-of-sample predictive accuracy under defensible validation protocols, and improvements are demonstrated relative to transparent, commensurate baseline models evaluated under identical spatially blocked cross-validation splits. This benchmarking strategy aligns with forecasting practice as discussed in Barkan et al. [52], where gains must be shown against simple and interpretable baselines while ensuring coherence across aggregation levels. In this study, we therefore evaluate performance at the edge level (primary target), examine regional aggregation under spatial cross-validation, and verify that improvements at the segment level translate into consistent aggregate patterns.

The framework has several practical implications. By substituting hand-specified, rule-based calculations with a data-driven predictive mapping, Mukara captures nonlinear interactions between external determinants and observed traffic volumes within a unified end-to-end modelling framework. At the same time, its input structure is aligned with the FSM tradition, which supports planning use cases where external scenarios (e.g., changes in population, employment, or land use) are available but historical traffic measurements may be sparse or unavailable. Although not tested outside the UK in this study, the design supports prediction on road segments with no prior flow observations, provided that comparable external features and network representations can be constructed. This property is relevant for data-sparse contexts. More generally, models that rely purely on historical data can struggle to anticipate network changes driven by new infrastructure, as illustrated by the Shenzhen–Zhongshan Link: while it alleviated congestion on the Humen and Nansha Bridges, it was associated with severe congestion within Shenzhen due to increased inflows to the city network [58].

Several limitations should be acknowledged. First, while Mukara is conceptually aligned with FSM logic, we did not include external models for direct comparison under identical data and assumptions. No existing deep learning method directly matches the present task setting—predicting highway-level daily volumes using external drivers in an FSM-like input format without historical traffic series—and implementing a traditional FSM on the UK network would require OD estimation and extensive calibration beyond the raw inputs used here. Without such tuning, FSM implementations can perform poorly in practice and would not provide a meaningful benchmark under the same assumptions. For this reason, we focus benchmarking on both statistical baselines and internal neural ablation variants evaluated under identical data splits and metrics, and on comparisons with published results that use different data, study designs, and evaluation settings.

Second, the study is predictive rather than causal. It does not attempt to identify exogenous effects of population, employment, land use, or network characteristics, and it does not resolve econometric identification concerns such as simultaneity, omitted variables, or reverse causality [59–61]. These variables co-evolve with transport systems over long time horizons, and Mukara should be interpreted as predicting realised traffic volumes conditional on observed spatial and socioeconomic configurations. Relatedly, the use of a static OSM snapshot for a multi-year traffic panel introduces temporal misalignment and potential measurement error. Although major road hierarchies and national-scale land-use structures in the UK evolve relatively slowly, this choice remains a pragmatic trade-off, and time-varying land-use and infrastructure datasets would be preferable where available.

Third, several modelling and data choices constrain performance and generalisability. Errors are higher for sensors with very low or very high volumes, which may reflect measurement noise, capacity constraints, junction effects, and the absence of time-varying operational drivers (e.g., incidents, weather, roadworks) that can be influential for extremes. In addition, sensor selection was designed to represent interurban trunk-road conditions with reliable coverage, including prioritising sensors near segment midpoints to reduce local access effects. While this reduces noise, any selection strategy may affect representativeness, and expanding coverage by matching more sensors to segments may improve training signal. Although the effective sample includes repeated edge–year observations, the number of distinct monitored segments remains a constraint when learning generalisable network-wide representations.

Finally, while Mukara supports spatial transfer to unmonitored links and regions within the studied network, external validation in a different geographic region or institutional context was not conducted. Transfer to regions with different modal structures, land-use patterns, road hierarchies, or data quality therefore remains an open empirical question in this paper.

These limitations motivate several directions for future research. A first practical step is to expand training coverage by automatically matching all available sensors to road segments rather than manually selecting a single representative sensor per segment, thereby increasing the number of targets and capturing within-segment variability. A second direction is to diagnose which parts of the FSM the model approximates well and where the main bottlenecks lie. For example, Deep Gravity has shown that OD distribution can be predicted using external determinants [31], suggesting that remaining challenges may be more related to modal split and assignment-like behaviour. Controlled experiments using synthetic or semi-synthetic data could support systematic evaluation of deep learning alternatives for individual FSM steps, or combinations of steps, under known ground truth. A third direction is architectural refinement. CNNs provide a convenient mechanism for aggregating gridded spatial context, but alternative spatial encoders (e.g., Vision Transformers) may better represent multi-scale or long-range spatial structure. Likewise, while GNNs are effective for information propagation, assignment-like behaviour may benefit from architectures that allow more interpretable interactions between embeddings, potentially integrating explicit impedance representations with structured attention or routing-inspired modules.

A broader development agenda includes (a) computational efficiency and suitability for real-time deployment, (b) extension to urban contexts, (c) long-term forecasting, (d) finer temporal resolutions such as daily or hourly prediction, (e) integration of uncertainty estimation through probabilistic modelling, and (f) transferability to entirely new regions with minimal adaptation. The current implementation is computationally lightweight, with memory usage peaking at around 11 GB. Training takes approximately 2 minutes per epoch (or about 15 seconds per yearly time step), and inference over the full network can be completed within about 5 seconds under the current setup. This runtime profile supports practical deployment and becomes increasingly relevant if the model is extended to higher temporal resolutions.

Extending Mukara to urban contexts is theoretically feasible but more challenging due to dense networks, frequent intersections, and stronger interactions with public transport systems. Addressing these complexities may require additional datasets such as public transport supply, signal timing, and richer operational information. For forecasting, the current annual-resolution model can generate scenario-based projections if future external drivers (e.g., population and employment forecasts) are available, but extrapolation far beyond the training range should be interpreted cautiously. Refining the temporal resolution to daily or hourly predictions would require modelling temporal dynamics (seasonal, weekly, diurnal cycles) and incorporating dynamic drivers such as weather, holidays, special events, and road maintenance logs. Adding static modal context (e.g., public transport availability, schedules, and costs) may further improve realism in multimodal settings.

We also tested an uncertainty-aware extension using heteroscedastic regression with a Gaussian negative log-likelihood loss to estimate both mean and variance. This variant produced weaker point prediction performance and less stable convergence, with minimum MAE increasing from roughly 9,000 to around 12,000. These results were therefore not included in the main evaluation. Nonetheless, uncertainty estimation remains important for planning and risk-aware applications, and alternative approaches such as Bayesian neural networks or ensemble methods may provide better-calibrated predictive intervals while preserving point accuracy.

Regarding transferability, Mukara can be applied to road segments with no historical traffic observations because it relies on external determinants rather than lagged traffic states. The attention-based message passing supports generalisation across network structures when comparable features are available. In practice, a lightweight fine-tuning procedure using a small amount of local data may help capture regional differences while retaining the advantages of minimal data requirements. However, full transfer to regions with distinct cultural, infrastructural, or institutional contexts remains to be evaluated.

Finally, issues of welfare analysis, economic efficiency, and market failure identification, while important, fall outside the scope of this study. Mukara is not intended to evaluate optimality or efficiency of observed traffic patterns, but to predict realised traffic volumes conditional on existing spatial and socioeconomic configurations. Extending the framework toward welfare-aware or policy-evaluative applications would be a valuable direction for future research.

## Conclusion

This study proposes Mukara, an end-to-end deep learning framework for predicting weekday daily traffic volumes on highway trunk road segments using only external socioeconomic, land-use, and network-related features. Using the UK trunk-road network as a case study, Mukara achieved a mean test MGEH of 50.74 and a mean test *R*^2^ of 0.583. These results are comparable to, and in some cases outperform, existing studies conducted under different settings, while addressing a more restrictive prediction task that excludes historical traffic observations. Ablation experiments showed that accurate prediction depends on the joint modelling of spatial context and network structure, with land-use features playing a particularly important role.

Mukara is intended as a predictive, planning-oriented framework. Within this scope, the results demonstrate that an integrated, representation-learning approach can approximate key elements of the demand-to-flow relationship without explicitly modelling individual steps of the four-step framework or requiring extensive calibration.

Future research could extend this framework by expanding sensor-to-segment matching to increase training coverage, incorporating time-varying spatial and network attributes, and improving the treatment of very low and very high traffic volumes. Additional work is also needed to evaluate transferability across different geographic and institutional contexts and to assess the performance of the approach under alternative temporal resolutions or modelling assumptions.

## Supporting information

S1 File Supplementary appendix (PDF). Contains Appendix S1–S8. Appendix S1 summarises the four-step travel demand model (FSM). Appendix S2 describes the sensor selection procedure, and Appendix S3 evaluates the representativeness of selected sensors. Appendix S4–S6 present robustness and sensitivity analyses, including the OSM vintage robustness test, weekend and holiday inclusion sensitivity, and GEH loss sensitivity analysis. Appendix S7 details the specification of baseline models, and Appendix S8 reports hierarchical aggregation consistency and planning-scale evaluation results.
(PDF)
