# Supplementary material for: Mukara: A deep learning alternative to the four-step travel demand model with a case study on interurban highway traffic prediction in the UK
Source: PLoS One. 2026 Apr 16;21(4):e0345576. doi: 10.1371/journal.pone.0345576 (PMC13086353; doi:10.1371/journal.pone.0345576)
Supplement: S1 File — Contains Appendix S1–S8. Appendix S1 summarises the four-step travel demand model (FSM). Appendix S2 describes the sensor selection procedure, and Appendix S3 evaluates the representativeness of selected sensors. Appendix S4–S6 present robustness and sensitivity analyses, including the OSM vintage robustness test, weekend and holiday inclusion sensitivity, and GEH loss sensitivity analysis. Appendix S7 details the specification of baseline models, and Appendix S8 reports hierarchical aggregation consistency and planning-scale evaluation results. (PDF) [file pone.0345576.s001.pdf]

Supplementary Information

S1 File. Supplementary Appendix for:
Mukara: a deep learning alternative to the four-step travel demand model with a case study on interurban highway traffic prediction in the UK

Yue Li[1], Shujuan Chen[1], Ying Jin[1]
[1]Martin Centre for Architectural and Urban Studies, University of Cambridge, 1–5 Scroope Terrace, Cambridge CB2 1PX, United Kingdom

Table of Contents

Appendix S1 Summary of the four-step travel demand model (FSM) 2
Appendix S2 Sensor selection procedure 4
Appendix S3 Representativeness check of selected sensors 6
Appendix S4 OSM vintage robustness check 8
Appendix S5 Weekend/holiday inclusion sensitivity 9
Appendix S6 GEH loss sensitivity analysis 10
Appendix S7 Methods of baseline models 12
Appendix S8 Hierarchical aggregation consistency and planning coherence 14

S1 Summary of the four-step travel demand model (FSM)

The classical four-step travel demand model (FSM) decomposes aggregate travel demand estimation into four sequential components: trip generation, trip distribution, modal split, and traffic assignment. Each step addresses a distinct behavioural or network-related mechanism and produces outputs that serve as inputs to subsequent stages. The following subsections provide a concise conceptual and mathematical overview of each component. Notation is defined locally to ensure this appendix remains self-contained.

S1.1 Trip generation

Trip generation estimates the number of trips produced and attracted by each spatial unit (zone) within the study area. Let i index zones. The number of trips generated by zone i, denoted T_i, is typically modelled as a function of socioeconomic and land-use characteristics. A common specification is a linear regression form:

$$T_i = \beta_0 + \sum_{m=1}^{M} \beta_m X_{im},$$

where X_{im} represents the m-th explanatory variable for zone i (e.g., population, household size, car ownership, employment), and β_m are estimated parameters. The output of this step is typically divided into trip productions (originating trips) and trip attractions (destination-bound trips), often denoted O_i and D_i, respectively. These aggregate trip totals form the basis for subsequent spatial allocation.

S1.2 Trip distribution

Trip distribution allocates the zonal productions O_i to destination zones j, producing an origin–destination (OD) matrix T_{ij}, where T_{ij} denotes the number of trips from origin zone i to destination zone j. A widely used formulation is the gravity model:

$$T_{ij} = A_i O_i B_j D_j f(C_{ij}),$$

where D_j represents destination attraction, C_{ij} denotes generalized travel cost between i and j (e.g., time or monetary cost), and $f(\cdot)$ is an impedance function, often exponential or power-decay. The terms A_i and B_j are balancing factors ensuring that row and column totals satisfy the constraints $\sum_j T_{ij} = O_i$ and $\sum_i T_{ij} = D_j$. The resulting OD matrix describes the spatial structure of travel demand prior to mode choice and network assignment.

S1.3 Modal split

The modal split step determines the proportion of trips between each origin–destination pair that are undertaken using different transport modes. Let k index available modes (e.g., car, bus, rail). A common behavioural formulation is the multinomial logit model:

$$P_{ij}^k = \frac{\exp(-\beta C_{ij}^k)}{\sum_{k'} \exp(-\beta C_{ij}^{k'})},$$

where P_{ij}^k is the probability that a trip between i and j uses mode k, C_{ij}^k is the generalized cost of mode k between i and j, and β is a cost sensitivity parameter. The mode-specific OD flow is then given by $T_{ij}^k = P_{ij}^k T_{ij}$. This step introduces behavioural modelling by linking travel choice to perceived costs and attributes of competing modes.

S1.4 Traffic assignment

Traffic assignment allocates mode-specific OD flows onto specific routes in the transport network. Let A and B denote alternative routes connecting a given OD pair. Under the user equilibrium principle (Wardrop's first principle), flows are distributed such that no traveller can reduce their travel cost by unilaterally changing routes. Formally, equilibrium requires that all used routes between an OD pair have equal and minimal travel cost:

$$C_A = C_B \quad \text{for all used routes } A, B.$$

Here, C_A and C_B denote the generalized travel costs associated with routes A and B, respectively. The output of this step consists of link-level traffic flows, travel times, and congestion patterns across the network. Assignment methods range from all-or-nothing allocation to iterative user equilibrium algorithms that account for congestion-dependent travel times.

Together, these four components form the traditional FSM pipeline, translating zonal characteristics into network-level traffic flows through a sequence of aggregate modelling stages.

S2 Sensor selection procedure

The representative-sensor selection procedure is illustrated in Figure S2. For each modelled road segment, all mapped candidate sensors were first retrieved and filtered using topology-based rules to remove slip-road sensors and those located entirely within junction influence zones. Remaining candidates were then ranked according to data availability metrics, and one representative sensor per segment was selected using an availability-first rule.

To ensure transparency, Table S2.1 reports the number of sensors retained and excluded at each stage of the procedure. Of the 3,972 candidate sensors initially mapped to the 498 modelled segments, 1,157 were removed during topology-based filtering. The remaining 2,815 sensors were evaluated based on temporal coverage, from which one representative sensor per segment was retained. In rare cases where no eligible candidate remained, an opposite-direction proxy was used (3 segments, approximately 0.6%). This stepwise accounting complements the workflow diagram and provides a quantitative summary of the selection mechanism.

Table S2.1: **Counts at each sensor filtering and selection step.**

**Step**	**Remaining sensors**	**Excluded at step**	**Notes**
Retrieve all candidate sensors mapped to the 498 modelled segments	3,972	–	All raw candidate sensors prior to topology filtering
Remove non-representative sensors based on topology rules	2,815	1157	Removes slip/ramp sensors and sensors fully within junction influence zones
Select representative sensor per segment (availability-first rule)	498	2,317	One sensor retained per segment; remaining candidates discarded
Fallback: opposite-direction proxy where no eligible sensor remains	3	–	Applied to 3 of 498 segments (≈0.6%)
**Final**	**498**	**3,486**	Total discarded sensors across all steps

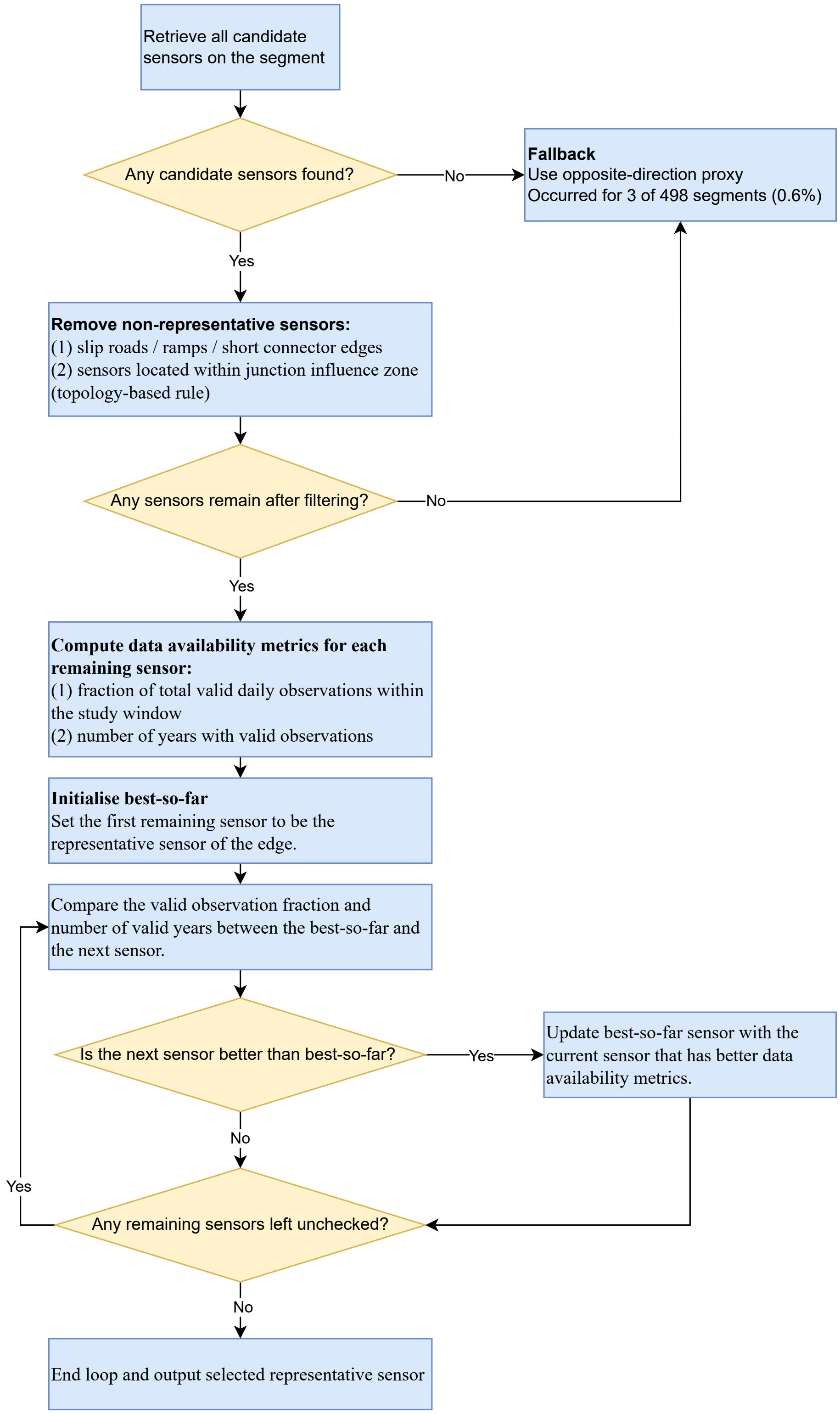

Figure S2: Workflow for selecting a representative traffic sensor for each road segment.

S3 Representativeness check of selected sensors

Table S3.1 summarises the quantitative representativeness of the selected (included) sensors compared with discarded candidate sensors. The included set contains one representative sensor per modelled road segment ($N = 498$), while discarded sensors comprise all other candidate sensors mapped to the same segments ($N = 3{,}486$). Regional distributions indicate that selected sensors are broadly distributed across England, with edge counts reflecting the density of the strategic road network in each region. As expected, discarded sensors are more concentrated in metropolitan regions such as London and the South East, where higher junction density and multi-lane configurations lead to multiple co-located sensors on the same segment.

Table S3.1: **Quantitative representativeness summary for traffic sensors: selected (included) vs discarded (excluded) candidate sensors.** The included set contains one representative sensor per modelled road segment ($N = 498$). Temporal coverage is the fraction of valid weekday daily observations within the 8-year study window (excluding holidays).

**Characteristic**	**Included sensors**	**Discarded sensors**
**Total sensors,** N	498 (100%)	3,486 (100%)
**Region (count, %)**		
North East	24 (4.8%)	86 (2.5%)
North West	64 (12.9%)	338 (9.7%)
Yorkshire and The Humber	60 (12.0%)	437 (12.5%)
East Midlands	88 (17.7%)	431 (12.4%)
West Midlands	76 (15.3%)	527 (15.1%)
East of England	58 (11.6%)	359 (10.3%)
London	32 (6.4%)	520 (14.9%)
South East	60 (12.0%)	568 (16.3%)
South West	36 (7.2%)	220 (6.3%)
**Road type (count, %)**		
Motorway	300 (60.2%)	1,992 (57.1%)
Trunk road	198 (39.8%)	1,494 (42.9%)
**Sensor position type (count, %)**		
Mainline	498 (100.0%)	2,329 (66.8%)
Slip/ramp	0 (0.0%)	481 (13.8%)
Junction influence zone	0 (0.0%)	676 (19.4%)
**Observed volume (mean, SD)**	33,735 (21,999)	30,151 (27,358)
**Temporal coverage (mean, SD)**	0.86 (0.10)	0.31 (0.24)

Road-type proportions remain comparable between included and discarded sets, with motorways accounting for approximately 60% of selected sensors. In contrast, sensor position types differ more substantially: 99% of selected sensors are located on mainline carriageways, while discarded sensors include a larger proportion of slip-road and junction-influence-zone sensors. This reflects the topology-based filtering criteria described in Section S2.

Differences are also observed in temporal coverage. Selected sensors exhibit substantially higher data completeness (mean coverage 0.86, SD 0.10) compared with discarded sensors (mean 0.31, SD 0.24), confirming that the selection rule primarily filters on availability rather than geographic location. Mean observed volumes are broadly comparable between the two sets, indicating that the

procedure does not disproportionately remove either low- or high-volume segments.

We also conducted a visual inspection of the spatial representativeness of the selected sensors. As depicted in Figure S3, the left panel shows segment-level volumes obtained by aggregating information from all sensors mapped to the same edge, with smoothing applied where multiple sensors were present in order to obtain a continuous spatial surface. This panel is presented solely to illustrate the overall spatial distribution of traffic intensity when no representative-sensor filtering is imposed. The right panel displays volumes derived from the selected representative sensor for each segment following the procedure described in Section S2. These values correspond to the observations used throughout the modelling framework.

Visual inspection indicates that the large-scale spatial structure of traffic demand on the entire strategic highway network remains consistent across both representations. This comparison demonstrates that the coverage-based representative-sensor selection procedure preserves the national spatial pattern of traffic volumes and does not introduce systematic spatial distortion.

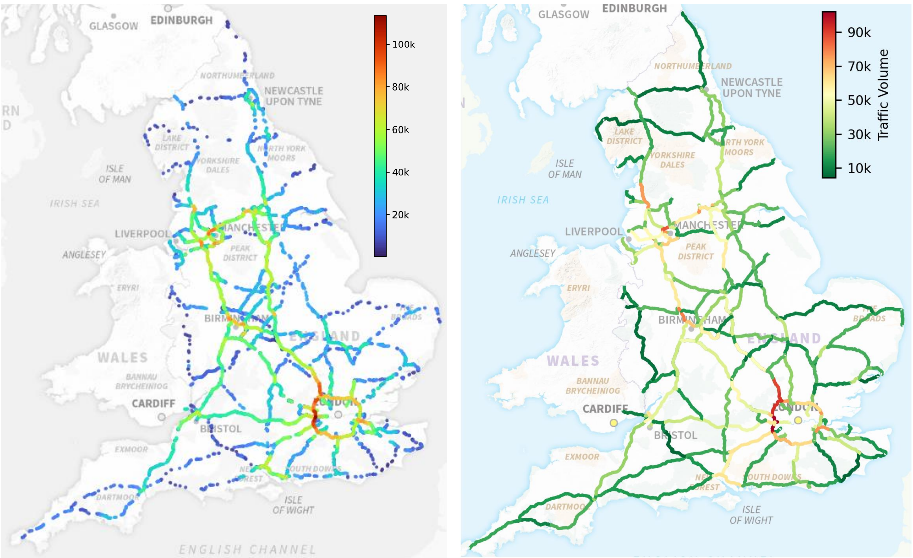

Figure S3: Spatial comparison between traffic volumes derived from (left) all available sensors and (right) the representative sensor selected for each road segment and used in this study. Basemap data: © Crown copyright and database right 2025 Ordnance Survey (OS OpenData), licensed under the Open Government Licence v3.0. Traffic data visualisation and analysis by the authors.

S4 OSM vintage robustness check

To evaluate the potential impact of temporal misalignment introduced by using a single OSM snapshot for all study years, we conducted a robustness analysis using an alternative historical OSM extract (timestamp: 2019-01-01). This snapshot was selected to be temporally closer to the early portion of the 2015–2022 study period.

All downstream preprocessing steps were repeated to ensure full consistency. Specifically, we re-rasterised land-use features, and extracted POI density layers using the identical pipeline applied to the 2023-01-01 snapshot. The resulting grid-based and segment-level features were then reconstructed and supplied to Mukara without any architectural modification.

The model was retrained and evaluated under the same spatially blocked 9-fold cross-validation protocol described in the main text. Table S4.1 reports aggregate predictive performance across folds for both OSM vintages.

Table S4.1: **Comparison of predictive performance under alternative OSM snapshots (spatially blocked 9-fold CV).** Values are reported as mean (standard deviation) across folds.

OSM snapshot	MGEH	MAE	$\mathbf{R^2}$
2023-01-01 (main)	57.63 (3.42)	9955 (612)	0.521 (0.072)
2019-01-01	58.14 (3.57)	10021 (648)	0.517 (0.075)

The differences in aggregate metrics are small: mean MGEH increases by 0.51, MAE increases by 66 vehicles per day (approximately 0.7% relative change), and R^2 decreases by 0.004. These variations fall within fold-level variability and do not materially affect conclusions. To conclude, the consistency of results across OSM vintages suggests that Mukara's predictive behaviour is not materially sensitive to moderate differences in snapshot timing. While local edits and POI updates occur continuously in OSM, the macro-scale structural and land-use features relevant to highway trunk-road demand appear sufficiently stable over the study period. These findings support the validity of using a single harmonised OSM snapshot for multi-year modelling in this context.

S5 Weekend/holiday inclusion sensitivity

In the main analysis, the target variable is defined as mean weekday daily traffic volume in order to focus on structurally stable commuting and inter-urban demand patterns. To assess whether excluding weekends and public holidays materially affects model performance, we conducted a sensitivity analysis in which Mukara was re-trained using mean daily traffic volumes computed across all available days (including weekends and public holidays). All preprocessing steps were repeated to ensure consistency. The model architecture and hyperparameters were unchanged. Training and evaluation were performed under the same spatially blocked 9-fold cross-validation protocol described in the main text.

Table S5.1 reports aggregate out-of-sample performance under the weekday-only specification (main analysis) and the all-days specification. Including weekends and public holidays leads to marginally improved aggregate metrics, with a reduction in both MGEH and MAE and a small increase in R^2. This modest improvement likely reflects the smoothing effect introduced by averaging across a larger number of days, which reduces day-to-day volatility in observed counts.

Table S5.1: **Comparison of predictive performance under weekday-only versus all-days specification (spatially blocked 9-fold CV).** Values are reported as mean (standard deviation) across folds.

**Specification**	**MGEH**	**MAE**	$\mathbf{R^2}$
Weekday-only (main)	57.63 (3.42)	9955 (612)	0.521 (0.072)
All days included	56.48 (3.27)	9734 (589)	0.533 (0.069)

To conclude, the results indicate that restricting the analysis to weekday averages does not materially affect predictive performance or alter substantive conclusions. The relative performance ranking of models remains unchanged, and spatial generalisability is preserved. The weekday-only specification is therefore retained in the main analysis to maintain conceptual alignment with standard transport planning practice, while the all-days specification is reported here for completeness and robustness.

S6 GEH loss sensitivity analysis

To assess the sensitivity of Mukara to the choice of training objective, we re-trained the full model using alternative regression losses, including mean squared error (MSE), mean absolute error (MAE), and Huber loss. All sensitivity experiments follow the same spatially blocked 9-fold cross-validation protocol as the main analysis (region-based hold-out), with hyperparameters tuned within training folds only. Models are evaluated using the same out-of-sample metrics reported in the main text (MGEH, MAE, and R^2), computed on held-out regions.

Table S6.1 summarises aggregate performance across folds. Overall, the headline metrics are broadly consistent across loss functions, indicating that Mukara's predictive capacity is not materially altered by the training objective. Models trained with MSE achieve slightly lower MAE and marginally higher R^2 on average, while models trained with MGEH maintain slightly lower MGEH. Huber loss yields intermediate behaviour.

Table S6.1: **Overall sensitivity to training objective under spatially blocked 9-fold CV.** Values are reported as mean (standard deviation) across folds.

**Training objective**	**MGEH**	**MAE**	$\mathbf{R^2}$
MGEH (main)	57.63 (3.42)	9955 (612)	0.521 (0.072)
MSE	58.12 (3.61)	9901 (648)	0.523 (0.070)
MAE	58.64 (3.74)	9843 (669)	0.519 (0.074)
Huber	57.91 (3.55)	9886 (631)	0.522 (0.071)

Because GEH incorporates an implicit volume-dependent weighting, we additionally report stratified performance by traffic-volume regime to diagnose whether different objectives preferentially improve low- or high-volume segments. Following the main analysis, sensors are grouped into quartiles based on their mean weekday daily traffic volume. Table S6.2 compares the two most contrasting objectives (MGEH vs MSE). Consistent with the theoretical weighting properties, MSE-trained models show slightly improved fit for the highest-volume quartile (Q4), whereas MGEH-trained models provide better proportional accuracy for the lowest-volume quartile (Q1). Differences in the middle quartiles are small.

Table S6.2: **Stratified sensitivity by traffic-volume quartiles under spatially blocked 9-fold CV.** Values are reported as mean (standard deviation) across folds. Quartiles are defined by sensor mean weekday daily traffic volume.

**Quartile**	**Training objective**	**MGEH**	**MAE**
Q1 (lowest volume)	MGEH	51.5 (5.2)	3248 (418)
	MSE	55.9 (5.6)	3412 (436)
Q2	MGEH	38.7 (3.9)	6894 (547)
	MSE	39.8 (4.1)	6812 (566)
Q3	MGEH	41.4 (3.6)	10638 (702)
	MSE	42.0 (3.8)	10554 (721)
Q4 (highest volume)	MGEH	69.8 (6.3)	19482 (1245)
	MSE	67.2 (6.6)	18694 (1312)

Taken together, these results indicate that the choice of training objective primarily redistributes error emphasis across traffic regimes rather than changing overall predictive capacity. We therefore

retain mean MGEH as the primary objective to align with established traffic-modelling validation practice and to promote balanced performance across heterogeneous traffic volumes, while reporting MSE/MAE/Huber sensitivity results here for transparency.

S7 Methods of baseline models

This section describes the additional commensurate baseline benchmarks introduced to complement the ablation experiments. All baseline models were trained and evaluated under the same spatially blocked cross-validation protocol as Mukara and assessed using identical performance metrics. To ensure comparability, all baselines used the same segment-level feature construction pipeline unless otherwise stated. Notation follows Table 2 in the main manuscript.

S7.1 Regularized linear regression (Ridge; L2 penalty)

A log-linear ridge regression model was fitted using the same segment-level raw feature vectors $\mathcal{X} = \{\mathbf{x}_{e_{ij}}\}$ as Mukara. For each directed edge $e_{ij} \in \mathcal{E}$ in the highway graph $\mathcal{G} = (\mathcal{V}, \mathcal{E})$, the feature vector $\mathbf{x}_{e_{ij}}$ includes: (i) $D_e = 5$ intrinsic edge features (e.g., length, road class, speed limit), and (ii) node-level contextual aggregates constructed at both endpoints v_i and v_j.

For each endpoint node, we aggregated $D_m = 106$ grid-based channels from $\mathcal{M}$ within a 21 km bounding box, corresponding to the best-performing configuration in Mukara. Consequently, the dimensionality of $\mathbf{x}_{e_{ij}}$ is

$$D_e + 2D_m = 5 + 2 \times 106 = 217.$$

Let $y_{t,e_{ij}} \in \mathcal{Y}$ denote the observed weekday daily traffic volume for edge e_{ij} in year t. For notational simplicity, we suppress the time index t in this section and write $y_{e_{ij}}$. The dependent variable was log-transformed to stabilise variance.

The ridge regression model is defined as:

$$\log y_{e_{ij}} = \beta_0 + \mathbf{x}_{e_{ij}}^\top \boldsymbol{\beta} + \varepsilon_{e_{ij}},$$

with parameters estimated by minimising the penalised least-squares objective:

$$\mathcal{L}_{\text{ridge}} = \sum_{e_{ij} \in \mathcal{T}} \left(\log y_{e_{ij}} - \beta_0 - \mathbf{x}_{e_{ij}}^\top \boldsymbol{\beta} \right)^2 + \lambda \|\boldsymbol{\beta}\|_2^2,$$

where $\mathcal{T}$ denotes the training subset within each spatial fold and λ is the L2 regularization parameter. The hyperparameter λ was selected exclusively within training folds to avoid information leakage.

S7.2 Gravity-interaction baseline (distance-decay gravity regression)

We implemented a classical gravity-style interaction baseline to provide a transparent and interpretable benchmark grounded in traditional spatial interaction modelling. Unlike the ridge regression, which uses the full 217-dimensional feature vector $\mathbf{x}_{e_{ij}}$, the gravity baseline compresses origin and destination characteristics into scalar mass terms. For each directed edge e_{ij} connecting nodes v_i and v_j, we define:

$$M_i = \text{aggregate mass at node } v_i, \quad M_j = \text{aggregate mass at node } v_j.$$

Consistent with gravity-model conventions, each mass is a single scalar composite. Specifically, we define:

$$M_i = \text{Population}_i + \text{Employment}_i,$$

where both quantities are aggregated from $\mathcal{M}_{pe}$ within the same 21 km bounding box used in the ridge specification. The same construction applies to M_j. Impedance between nodes v_i and v_j is

represented by travel time C_{ij} computed along the road network $\mathcal{G}$. A standard exponential decay function is applied:

$$f(C_{ij}) = \exp(-\gamma C_{ij}),$$

where γ is a decay parameter estimated from training data. The gravity interaction score for edge e_{ij} is then defined as:

$$G_{e_{ij}} = M_i M_j \exp(-\gamma C_{ij}).$$

Observed edge-level volume is linked to this gravity score through a log-linear specification:

$$\log y_{e_{ij}} = \alpha_0 + \alpha_1 \log G_{e_{ij}} + \varepsilon_{e_{ij}}.$$

Substituting the definition of $G_{e_{ij}}$, this is equivalent to:

$$\log y_{e_{ij}} = \alpha_0 + \alpha_1 (\log M_i + \log M_j - \gamma C_{ij}) + \varepsilon_{e_{ij}}.$$

Thus, compared with the ridge regression that utilises $\mathbf{x}_{e_{ij}} \in \mathbb{R}^{217}$, the gravity baseline reduces explanatory structure to three interpretable components: origin mass M_i, destination mass M_j, and travel-time impedance C_{ij}. The distance-decay term applies exclusively to the impedance variable.

S7.3 Random Forest regressor

A non-linear ensemble baseline (Random Forest) was trained using the same raw feature vectors $\mathbf{x}_{e_{ij}}$ as defined above. The response variable $y_{e_{ij}}$ was log-transformed for consistency with the linear specifications. The Random Forest approximates a non-parametric mapping:

$$\log y_{e_{ij}} = f(\mathbf{x}_{e_{ij}}),$$

where $f(\cdot)$ is an ensemble of decision trees constructed via bootstrap aggregation and random feature selection.

Hyperparameters, including the number of trees, maximum tree depth, and minimum samples per leaf, were tuned exclusively within the training folds of the spatially blocked cross-validation protocol. This ensures strict comparability with the ridge regression and Mukara models while preventing information leakage across folds.

S8 Hierarchical aggregation consistency and planning coherence

To assess planning relevance, we evaluate whether edge-level predictions aggregate coherently to policy-relevant totals at regional and national levels. Aggregated predicted totals are obtained by summing edge-level predictions within each region and across the entire network. These are then compared with aggregated observed totals, and absolute as well as percentage deviations are reported (Tables S8.1 and S8.2). Aggregation coherence is interpreted diagnostically: small percentage deviations indicate that the model does not introduce systematic bias when predictions are summed across spatial scales.

Table S8.1: **Hierarchical aggregation consistency under random cross-validation (test sets).** All totals are expressed in millions of weekday vehicle counts.

Region	Observed	Predicted	Abs. diff.	Error (%)
North East	0.728	0.722	0.006	-0.8%
North West	2.342	2.358	0.016	+0.7%
Yorkshire and The Humber	2.063	2.044	0.019	-0.9%
East Midlands	2.781	2.799	0.018	+0.6%
West Midlands	2.654	2.632	0.022	-0.8%
East of England	1.742	1.759	0.017	+1.0%
London	1.964	1.979	0.015	+0.8%
South East	1.897	1.884	0.013	-0.7%
South West	0.629	0.635	0.006	+1.0%
**National total**	**16.800**	**16.812**	**0.012**	**+0.07%**

Table S8.2: **Hierarchical aggregation consistency under spatial cross-validation (test sets).** All totals are expressed in millions of weekday vehicle counts.

Region	Observed	Predicted	Abs. diff.	Error (%)
North East	0.728	0.756	0.028	+3.8%
North West	2.342	2.296	0.046	-2.0%
Yorkshire and The Humber	2.063	2.128	0.065	+3.2%
East Midlands	2.781	2.719	0.062	-2.2%
West Midlands	2.654	2.732	0.078	+2.9%
East of England	1.742	1.703	0.039	-2.2%
London	1.964	1.856	0.108	-5.5%
South East	1.897	1.842	0.055	-2.9%
South West	0.629	0.667	0.038	+6.0%
**National total**	**16.800**	**16.699**	**0.101**	**-0.6%**

Under random cross-validation (Table S8.1), reported values represent the mean across folds. Because training and test edges are randomly distributed across regions, regional aggregates include contributions from both training and test samples in each fold. It is therefore expected that aggregated deviations are small. Indeed, regional percentage errors are generally below ±1%, and the national deviation is approximately 0.07%, indicating near-perfect aggregation consistency under this evaluation setting.

Spatial cross-validation (Table S8.2) provides a more stringent assessment, as entire regions are held out during training. In this setting, some regional deviations are observed, including modest underestimation in London and moderate overestimation in the South West. However, regional percentage errors remain within a narrow band (generally within $\pm 6\%$), and the national-level deviation is below 1%. These results are consistent with the expectation that aggregation reduces idiosyncratic edge-level noise: while individual segment predictions may vary, positive and negative deviations tend to cancel out when summed across regions. Overall, the model demonstrates coherent behaviour under both evaluation schemes, supporting its suitability for regional and network-scale planning analyses.
